# Compromised astrocyte function and survival negatively impact neurons in infantile neuronal ceroid lipofuscinosis

**DOI:** 10.1186/s40478-018-0575-4

**Published:** 2018-08-08

**Authors:** Jenny Lange, Luke J. Haslett, Emyr Lloyd-Evans, Jennifer M. Pocock, Mark S. Sands, Brenda P. Williams, Jonathan D. Cooper

**Affiliations:** 1 Department of Basic and Clinical Neuroscience, King’s College London, Institute of Psychiatry, Psychology & Neuroscience, Maurice Wohl Clinical Neuroscience Institute, 5 Cutcombe Road, London, SE5 9RX UK; 2 School of Biosciences, Cardiff University, Museum Avenue, Cardiff, CF10 3AX UK; 3 Department of Neuroinflammation, Institute of Neurology, University College London, 1 Wakefield Street, London, WC1N 1PK UK; 4 Departments of Medicine and Genetics, Washington University School of Medicine, 660 South Euclid Avenue, St. Louis, MO 63110 USA; 5 Department of Pediatrics, Los Angeles Biomedical Research Institute at Harbor-UCLA Medical Center, and David Geffen School of Medicine UCLA, 1124 West Carson Street, Hanley Hardison Building, Torrance, CA 90502 USA; 6 Department of Pediatrics, Washington University School of Medicine, Campus Box 8208, 660 S Euclid Avenue, St. Louis, MO 63110 USA

**Keywords:** Infantile batten disease, CLN1 disease, neuronal ceroid lipofuscinosis, Neuron-glial interactions, Astrocyte and microglial dysfunction

## Abstract

The neuronal ceroid lipofuscinoses (NCLs) are the most common cause of childhood dementia and are invariably fatal. Early localized glial activation occurs in these disorders, and accurately predicts where neuronal loss is most pronounced. Recent evidence suggests that glial dysfunction may contribute to neuron loss, and we have now explored this possibility in infantile NCL (INCL, CLN1 disease). We grew primary cultures of astrocytes, microglia, and neurons derived from Ppt1 deficient mice (*Ppt1*^*−/−*^) and assessed their properties compared to wildtype (WT) cultures, before co-culturing them in different combinations (astrocytes with microglia, astrocytes or microglia with neurons, all three cell types together). These studies revealed that both *Ppt1*^*−/−*^ astrocytes and microglia exhibit a more activated phenotype under basal unstimulated conditions, as well as alterations to their protein expression profile following pharmacological stimulation. *Ppt1*^*- /−*^ astrocytes also displayed abnormal calcium signalling and an elevated cytoplasmic Ca^2+^ level, and a profound defect in their survival. *Ppt1*^*−/−*^ neurons displayed decreased neurite outgrowth, altered complexity, a reduction in cell body size, and impaired neuron survival with prolonged time in culture. In co-cultures, the presence of both astrocytes and microglia from *Ppt1*^*−/−*^ mice further impaired the morphology of both wild type and *Ppt1*^*−/−*^ neurons. This negative influence was more pronounced for *Ppt1*^*−/−*^ microglia, which appeared to trigger increased *Ppt1*^*−/−*^ neuronal death. In contrast, wild type glial cells, especially astrocytes, ameliorated some of the morphological defects observed in *Ppt1*^*−/−*^ neurons. These findings suggest that both *Ppt1*^*−/−*^ microglia and astrocytes are dysfunctional and may contribute to the neurodegeneration observed in CLN1 disease. However, the dysfunctional phenotypes of *Ppt1*^*−/−*^ glia are different from those present in CLN3 disease, suggesting that the pathogenic role of glia may differ between NCLs.

**Electronic supplementary material:**

The online version of this article (10.1186/s40478-018-0575-4) contains supplementary material, which is available to authorized users.

## Introduction

The neuronal ceroid lipofuscinoses (NCLs, or Batten disease) are a large group of inherited lysosomal storage disorders, each caused by mutations in an individual gene [8, 32]. The age of onset and rate of disease progression are determined by which gene is deficient, and which mutation is present within that gene. The NCLs are collectively the largest cause of childhood dementia and share a range of similar clinical features, including loss of vision, epileptic seizures, and declines in mental and motor abilities, all invariably leading to premature death [2, 9, 50].

Despite recent advances in the treatment of late infantile NCL (CLN2 disease) via enzyme replacement therapy [30], the other forms of NCL all remain untreatable. This includes infantile NCL (INCL or CLN1 disease), an earlier onset and more rapidly progressing form of NCL, which is caused by a mutation in the *CLN1/PPT1* gene (Vesa et al., 1995; [5, 21]). However, it remains unclear how deficiency in palmitoyl protein thioesterase-1 (PPT1), the lysosomal enzyme encoded by this gene results in the devastating neurodegenerative impact upon the brain that typifies CLN1 disease.

The generation of an INCL mouse model (*Ppt1*^*−/−*^ mice) [17] has made it possible to study the consequences of PPT1 deficiency [3, 23, 29], and test a range of pre-clinical interventions [19]. These mice display a progressive CLN1 disease-like phenotype, with well-defined declines in behavioural and neurological performance, and degenerative changes that are most pronounced within the thalamocortical system and cerebellum. Characteristically these mice display a profound activation of both microglia and astrocytes, which precedes neuron loss [23, 29]. The regional localization of this glial activation correlates closely with distribution of subsequent neuron loss, leading to the suggestion that these events may be causally related.

Rather than their traditional supportive role, evidence is emerging that both astrocytes and microglia can directly contribute to neuron loss in a variety of disease states [25]. Amongst these are several lysosomal storage disorders [11, 41, 48], including the juvenile form of NCL, CLN3 disease or JNCL [35, 51]. We have recently shown via primary cultures derived from Cln3 deficient mice that astrocytes and microglia are both dysfunctional, and when co-cultured can harm healthy neurons and kill Cln3 deficient neurons, suggesting a direct influence of glia upon the pathogenesis of this form of NCL [35].

The extent of glial activation is much more pronounced in CLN1 disease, but as longitudinal studies show [23, 29], still accurately predicts the sites where neuron loss subsequently occurs. This raises the question of what the role of glia in this most profoundly neurodegenerative form of NCL may be. As a first step to resolving this question, we derived primary cultures of either astrocytes or microglia from *Ppt1*^*−/−*^ mice, and compared their properties and response to stimulation to those derived from wild type controls.

This in vitro study revealed a range of abnormal phenotypes different to those seen in Cln3 disease glia [35]. *Ppt1*^*−/−*^ astrocytes and microglia both appeared more activated than WT cells under basal unstimulated culture conditions. However, most notable was a profound defect in the survival of *Ppt1*^*−/−*^ astrocytes, which also displayed marked dysregulation of intracellular calcium handling, and subsequent death by apoptosis. Primary cortical neuron cultures from *Ppt1*^*−/−*^ mice displayed abnormal dendritic morphology and impaired survival, especially of interneurons. Using a co-culture system wild type glia ameliorated these neuronal phenotypes, whereas *Ppt1*^*−/−*^ astrocytes predominantly impaired the morphology of WT neurons, and *Ppt1*^*−/−*^ microglia appeared to trigger increased *Ppt1*^*−/−*^ neuronal death via apoptosis. These effects were more pronounced in mixed glial co-cultures when both *Ppt1*^*−/−*^ astrocytes and microglia were grown together with neurons. Taken together, these data suggest that the dysfunction of astrocytes and microglia in CLN1 disease is detrimental to neurons and may lead to their loss.

## Materials and methods

### Animals

Homozygous Ppt1 deficient (*Ppt1*^*−/−*^) mice were originally created through the deletion of the last exon in the *Ppt1* coding sequence [17], and were subsequently bred for at least 10 generations onto a C57/BL/6 J background [16]. The colony was maintained together with a colony of C57/BL/6 J wild type (WT) control mice originally derived from the crossing of heterozygous *Ppt1*^*+/−*^ mice. All animal maintenance and experimental procedures were carried out according to the UK Scientific Procedures (Animals) Act (1986).

### Tissue culture

#### Preparation of glial cultures

Mixed glial cells were isolated from post-natal day 1–5 (P1-P5) *Ppt1*^*−/−*^ or WT mouse cerebral cortices, as previously described [31, 35]. Cultures reached confluence after 10–14 days, at which point they were composed of a base layer of non-dividing astrocytes and an upper layer of microglia as well as a few oligodendrocytes. To generate astrocyte cultures, microglia were removed by shaking cultures at 180 rpm for 10–12 h in a humidified incubator (5% CO_2_, 37C, ([31]). The remaining astrocyte monolayer was treated with cytosine arabinoside (Ara-C) for 7 days to abolish any remaining dividing cells. Astrocytes for immunofluorescence staining experiments were plated at 1.5 × 10^4^ cells per well onto poly-D-lysine (PDL, 25μg/ml, Sigma) coated glass coverslips in 24 well plates. For all functional assays unless otherwise described, astrocytes were plated at 14.25 × 10^4^ cells per well onto PDL coated 6 well plates and kept in phenol-red-free DMEM supplemented with 4500 mg/L glucose (Sigma, Poole, UK), to prevent the presence of phenol red from providing a background interference.

#### Microglial cultures

Microglial cultures were isolated from mixed glial cultures using the shaking method described above [31, 35]. The harvested cells were resuspended in RPMI 1640 supplemented with 5% FBS, P/S, 2 mM L-Glutamine, macrophage colony-stimulating factor and granulate macrophage colony-stimulating factor, and consequently plated on PDL coated (as above) 1.5 cm coverslips placed in 24 well plates. They were maintained for 5 days, before growth factors were removed for a further two days. At the start of all experiments, all glial cultures had been maintained for approximately 21 days, yielding cultures of > 99% purity.

#### Microglia for co-cultures

Due to the difficulty of detaching microglia from PDL coated plastic surfaces, a different method was used to generate microglia for co-culturing with either astrocytes or neurons. These microglia were prepared from P3-P5 *Ppt1*^*−/−*^ or WT mouse cerebral cortices, initially generating mixed glial cultures as described above. Conditioned medium from these mixed cultures was collected and cultures subsequently incubated at 37 °C with 1 mL/well of Trypsin-EDTA (0.5mgml-1, Sigma). After 45 min the cell monolayer, consisting primarily of astrocytes, was removed along with the supernatant and discarded. The remaining microglial cells were washed before being incubated in a 1:1 mixture of conditioned and fresh medium. These microglial cultures were ready to be used in co-culture experiments after 48 h, when they were removed from wells using 0.5 ml Accutase (Sigma) and a cell scraper (Corning). Microglia were then added to co-cultures at a concentration of 50 × 10^4^ cells/ml per well.

#### Neuronal cultures

Neuronal cultures were generated from P0 *Ppt1*^*−/−*^ and WT mouse cortices as previously described [35] and plated on PDL-coated 13 mm coverslips placed in 24 well plates at a concentration of 15 × 10^4^ cells/cm^2^. These cultures were maintained for 7, 9 or 14 days.

#### Co-cultures

Neurons to be grown in co-cultures were plated onto 19 mm diameter coverslips in placed in 12 well plates at a concentration of 15 × 10^4^ cells/cm^2^. After 7 days, glial cells previously maintained in culture for 21 days were added to these neurons at a density of 100 × 10^4^ cells/ml for astrocytes or mixed glia, and 50 × 10^4^ cells/ml for microglia. Additional coverslips plated with just glia (either astrocytes, microglia or of mixed glia), or only neurons were set up as control conditions. Co-cultures were grown for an additional 2 or 7 days before commencing analysis, making the neuronal cultures used in these experiments 9 or 14 days old. The composition of 14 day old neuronal cultures was assessed using cell type-specific phenotypic markers, as described below.

##### Pharmacological activation of glial cells

Astrocytes were stimulated with lipopolysaccharide (LPS, 1 μg/ml LPS, Sigma) and interferon-gamma (IFN-γ, 100 U/ml, Thermo Scientific) for 24 or 48 h, while microglia were stimulated with LPS alone for either 6 or 24 h [35]. Mixed glial cultures were stimulated for 24 h using both stimuli.

##### Immunofluorescence staining

Cultures were fixed in 4% PFA and stained as previously described [35] and across all experiments, nuclei were counterstained with DAPI. Cell type-specific markers were used to assess culture composition, with glial fibrillary acidic protein (GFAP, Rabbit polyclonal, 1:500, Dako) being used to identify astrocytes, Cluster of Differentiation 68 (CD68, Rat monoclonal, 1:250, Serotec) to identify microglia, Map2 MAP2 (mouse monoclonal, 1:1000, Abcam) to identify neurons and O4 (Mouse monoclonal, 1:100, Covance) to identify oligodendrocytes. Cytoskeletal components were examined by staining cultures with Phalloidin, α- and β-tubulin (monoclonal and polyclonal antibodies respectively, both from Sigma and used at 1:1000). All secondary antibodies were used 1:1000 and obtained from Invitrogen. Coverslips were mounted using Fluoromount G and stained cells were visualized using a Zeiss *AxioImager Z1* microscope (Carl Zeiss, Ltd) and images captured with a monochrome *AxioCamMR3* camera, using *AxioVision* 4.8 Imaging software.

##### Morphological Assessment

Images of 10 random fields were taken per coverslip of GFAP stained, non-overlapping astrocytes and soma size was measured using *ImageJ* (National Institutes of Health, Bethesda, MD). Neuron soma size and neurite length were measured using *ImageJ* on images of 10 random fields per coverslip in neuronal cultures or 20 random fields per coverslip in co-cultures. The length of the longest neurite was obtained per neuron; as well as mean neurite length per neuron. The morphological response of microglia to stimulation was assessed by assigning cells to 3 sub categories; Type 1 microglia which exhibit extended processes and an elongated cell body, Type 2 microglia with retracted processes and a rounded cell body and Type 3 microglia exhibiting a very small, rounded cell body. 5 random fields of at least 200 cells were counted per coverslip per condition and the percentage of each subtype was determined [35].

##### Live/Dead Assay

Cell viability in WT and *Ppt1*^*−/−*^ astrocyte cultures was assessed using a Live/Dead kit (Invitrogen) according to the manufacturer’s instructions. Briefly, Live/Dead dye was diluted in DMSO and added 30 min prior to fixation of cultures 24, 48 or 72 h after plating astrocytes onto coverslips. Astrocyte cultures were then co-stained with GFAP and 10 random fields were counted per coverslip.

##### Calcium Measurements

Astrocytes were plated in μ-Slide 8 well imaging dishes (Sigma) at 1.5cells/cm^2^. Cells were loaded with 5 μM Fura2-AM (Teflabs) in DMEM with 1% BSA and 0.025% Pluronic acid F127 for 1 h at room temperature, before being washed in imaging buffer (HBSS, 1 mM HEPES and 1 mM MgCl_2_) and left at room temperature for 10 min. Intracellular Ca^2+^ concentrations and responses were recorded with a Colibri LED microscope system, using an Axiocam Mrm CCD camera and *Axiovision* software (Version 4.7) with an additional physiology module for live cell Ca^2+^ imaging (Zeiss). Recordings of the Ca^2+^ probe baselines were taken prior to agonist addition in order to provide basal Ca^2+^ measurements of the cell, and all agonist-induced responses were compared at peak height.

Release of endoplasmic reticulum (ER) Ca^2+^ was measured after addition of 5 μM of thapsigargin (Sigma). Lysosomal Ca^2+^ release was measured using a method adapted from [26]. Following with 5 μM ionomycin (Calbiochem) to prevent release from intracellular Ca^2+^ stores, the addition of 10 μM nigericin (Sigma) was used to induce Ca^2+^ release from the lysosome. To examine store operated Ca^2+^ entry, ER Ca^2+^ release was triggered as described, and following return to the baseline, 1 mM Ca^2+^ was added to the imaging medium. In order to trigger Ca^2+^ plasma membrane influx, 100 μM ATP (Sigma) was added after astrocytes were loaded with Fura2-AM in imaging buffer and 1 mM CaCl_2_.

##### Protein Secretion

To compare pharmacologically stimulated cytokine secretion by WT and *Ppt1*^*−/−*^ glia, supernatant was collected from mixed glial, astrocyte and microglial cultures and analysed with ELISA kits (Signosis, Santa Clara, CA). A mouse oxidative stress ELISA kit was selected to quantify microglial cytokine secretion based on previous evidence that *Ppt1*^*−/−*^ neurons exhibit signs of oxidative stress [49], a custom mouse cytokine ELISA kit was used to examine cytokine secretion by astrocytes based on pilot cytokine data generated in our laboratory, and cytokine secretion in mixed glial cultures was assessed with a mouse cytokine ELISA kit (all kits from Signosis). All these experiments were carried out according to the manufacturer’s instructions.

##### Statistical analysis

All data were collected into Microsoft Excel spreadsheets and analysed in Graphpad Prism. All data are presented as mean ± SEM. A student’s t-test was used to analyse data between two groups, data sets with more than two groups were analysed with a one-way ANOVA using Bonferroni’s correction. All experiments were repeated in triplicate, using three technical replicates per experiment, unless otherwise stated. Data were considered statistically significant if *p* ≤ 0.05, *p* values are marked * if *p* ≤ 0.05, ** if *p* ≤ 0.01 and *** if *p* ≤ 0.001.

## Results

### Ppt1^−/−^ astrocytes exhibit an activated phenotype under basal conditions

Astrocyte activation can be observed as early as 3 months of age in *Ppt1*^*−/−*^ mice, and progressively becomes more pronounced and widespread towards the later stages of the disease [23, 29]. Before comparing the in vitro phenotypes of *Ppt1*^*−/−*^ and WT astrocytes we first defined the cellular composition of our astrocyte cultures. One week after plating, over 99% of DAPI positive cells in astrocyte cultures of either genotype were positive for the astrocyte marker glutamine synthetase (GS) (WT: 99.2% GS + ve, 0.71% CD68 + ve, and 0% O4 + ve; *Ppt1*^−/−^: 99.6% GS + ve, 0.35% CD68 + ve, and 0% O4 + ve).

**Fig. 1:**
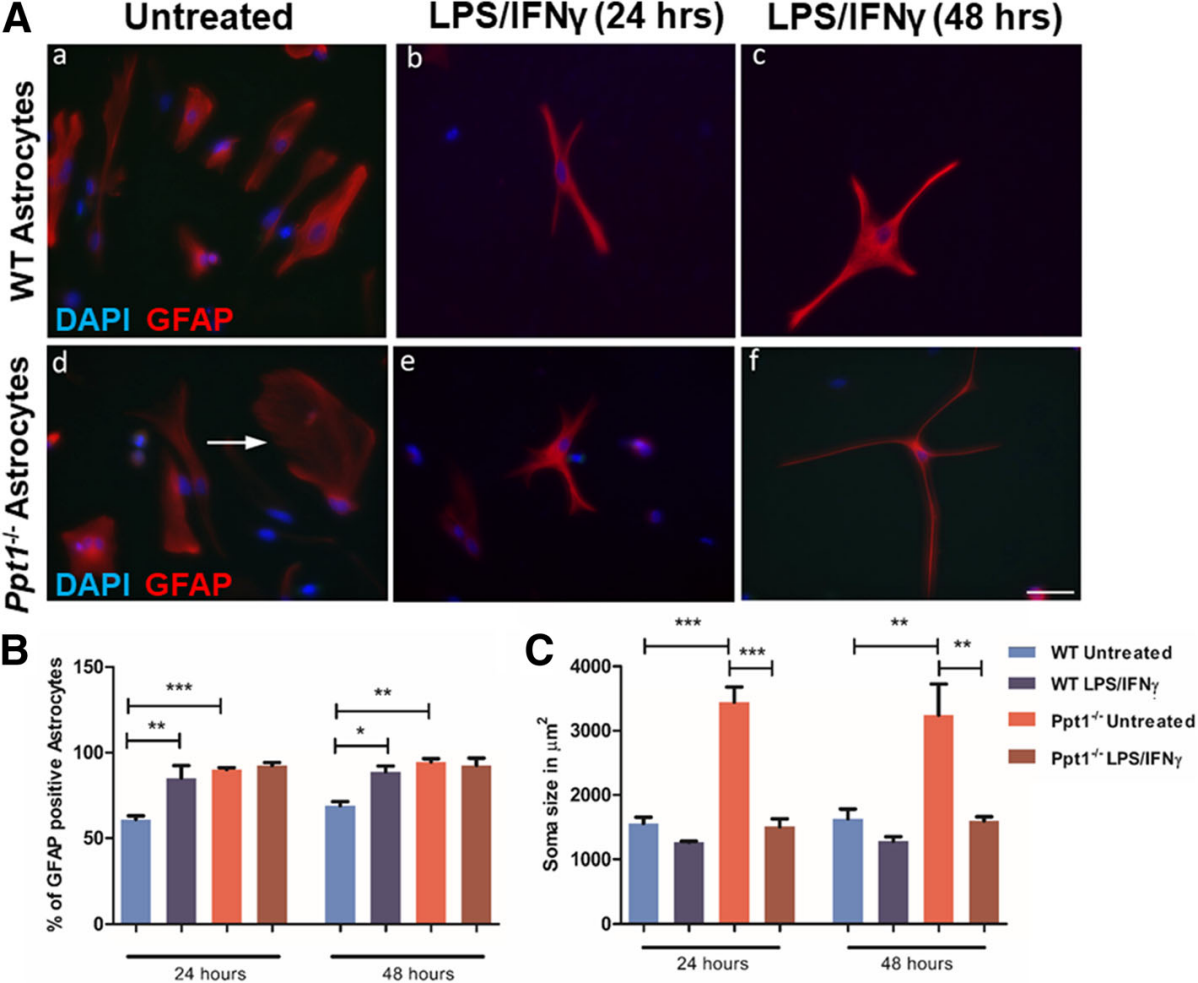
Ppt1 deficient *(Ppt1*^*−/−*^*)* astrocytes exhibit a reactive phenotype. **A** Wild type (WT) and *Ppt1*^*−/−*^ Astrocytes were stained for glial fibrillary associated protein (GFAP) to assess GFAP expression and astrocyte morphology under basal and stimulated (LPS/IFNγ) conditions after 24 and 48 h. Immunostaining for GFAP (red) revealed the morphology of *Ppt1*^*−/−*^ and WT astrocytes under basal and stimulated conditions, with *Ppt1*^*−/−*^ astrocytes exhibiting a large flattened morphology (white arrow) under basal conditions. **B** A higher percentage of *Ppt1*^*−/−*^ astrocytes stained positively for (GFAP) under basal conditions, with no significant upregulation following stimulation. **C** The soma size or spread of *Ppt1*^*−/−*^astrocytes was much larger under basal conditions than in WT astrocytes, however was reduced following stimulation for 24 and 48 h. (Data shown as Mean ± SEM using a one way ANOVA, *n* = 3). Scale bar = 50 μm

Using GFAP as a marker whose increased expression is associated with astrocyte activation, we first compared the morphology of astrocytes in cultures derived from mice of both genotypes, under both basal and simulated conditions (Fig. 1A). Under basal conditions the morphology of cultured *Ppt1*^−/−^ astrocytes was more heterogeneous than that seen in WT cultures, with many GFAP-positive astrocytes appearing larger in *Ppt1*^*−/−*^ cultures (Fig. 1A). The proportion of GFAP-positive cells under basal unstimulated conditions was significantly higher in *Ppt1*^*−/−*^ astrocyte cultures than in WT cultures (Fig. 1B). Cultures were then stimulated with LPS and IFNγ, to examine astrocyte response to a standardized pharmacological stimulus [35]. Although the proportion of GFAP-positive astrocytes was significantly upregulated in WT cultures after both 24 h and 48 h of stimulation, no further changes in the proportion of GFAP-positive astrocytes were observed in *Ppt1*^*−/−*^ cultures upon stimulation (Fig. 1B). These data reveal that although more *Ppt1*^*−/−*^ astrocytes express GFAP under basal culture conditions, this proportion does not increase further upon stimulation with LPS/ IFNγ, as it does with WT astrocytes.

To quantify the morphological responses of astrocytes to stimulation, we measured astrocyte soma size (Fig. 1C). Under basal conditions, *Ppt1*^−/−^ astrocytes exhibited a markedly and significantly larger soma size than WT astrocytes. However, upon stimulation for 24 or 48 h *Ppt1*^*−/−*^ astrocytes dramatically reduced their cell body size, a typical response of cultures astrocytes to pharmacological stimulation, so that they were now of a similar size to stimulated WT astrocytes at either time point (Fig. 1C). Taken together these data reveal that although appearing morphologically different to WT astrocytes under basal conditions, *Ppt1*^−/−^ astrocytes still respond robustly to pharmacological stimulation by changing their shape.

### Increased secretion of soluble factors by Ppt1^−/−^ astrocytes

**Fig. 2:**
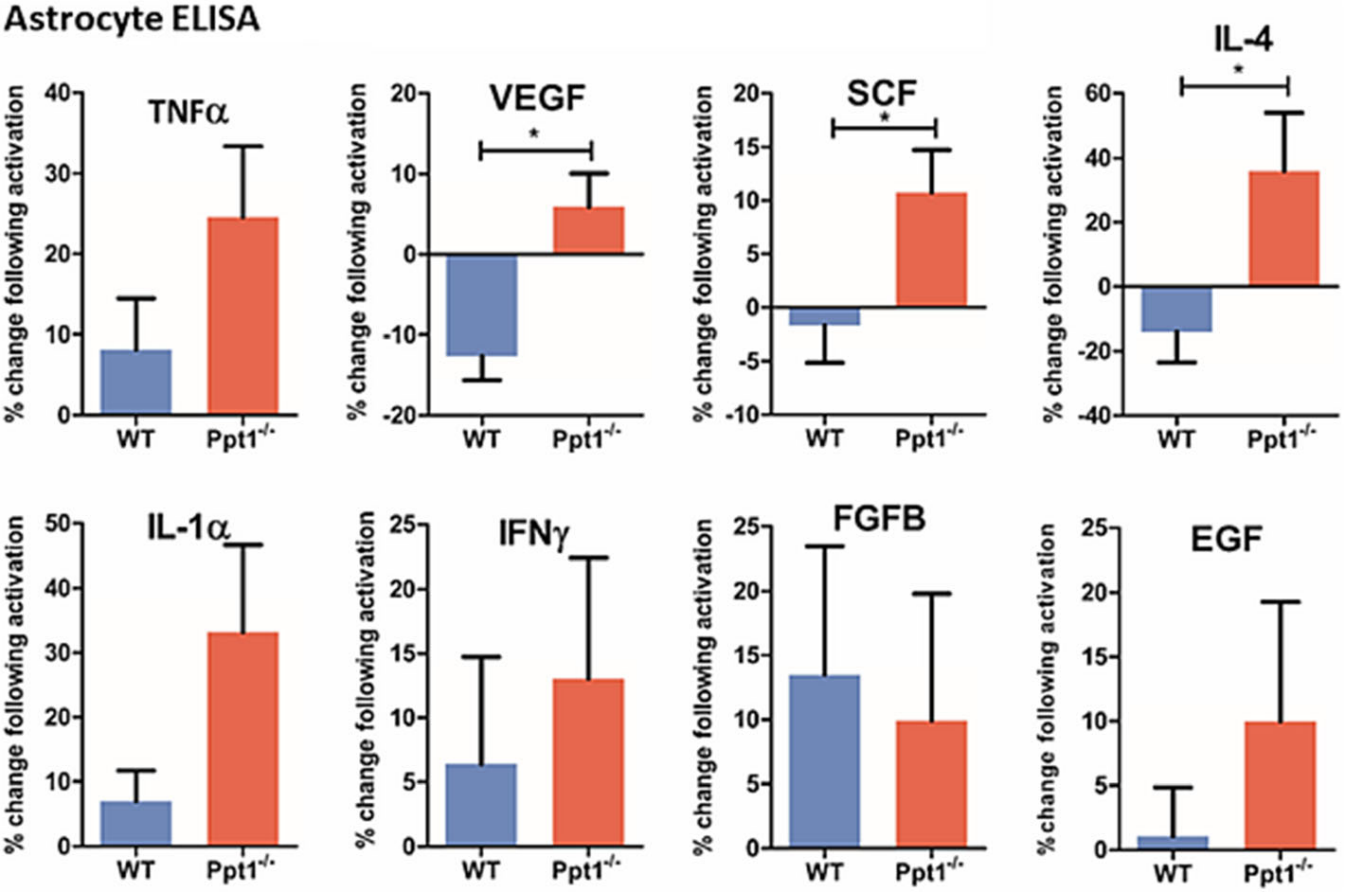
Expression of SCF, VEGF and IL-4 was significantly higher in Ppt1 deficient (*Ppt1*^*−/−*^) astrocytes after stimulation. Supernatant was collected from wild type (WT) and *Ppt1*^*−/−*^ astrocyte cultures kept under basal and stimulated conditions for 24 h. Cytokine release was assessed using an ELISA kit (Signosis) and calculating changes in expression between basal and stimulated conditions. No statistically significant changes were observed in the secretion of Epidermal growth factor (EGF), Interferon γ (IFNγ), Tumour necrosis factor α (TNFα), Expression of Basic flbroblast growth factor (FGFb) and Interleukin 1-α (IL1α). Stem cell factor (SCF), Vascular endothelial growth factor (VEGF) and Interleukin-4 (IL-4) was significantly higher in *Ppt1*^*−/−*^ astrocyte cultures following stimulation than in WT cultures. (Data shown as Mean ± SEM using a t-test, *n* = 3)

Increased secretion of specific soluble proteins is a hallmark of astrocyte activation (Lucas et al., 2006), and we used an ELISA kit to investigate the release of soluble factors following stimulation in our astrocyte cultures. No significant differences in the release of TNFα, IL-1α, IFNγ, FGFB, or EGF were observed between WT and *Ppt1*^*−/−*^ astrocytes following 24 h exposure to LPS and IFNγ (Fig. 2). However, significantly higher amounts of IL-4, SCF and VEGF were released by *Ppt1*^*−/−*^ astrocytes (35.98 ± 17.80%; 10.74 ± 3.98%; 5.90 ± 4.16% more, respectively) than by WT astrocytes (− 14.12 ± 9.39%; − 1.68 ± 3.47%; − 12.61 ± 3.02% less, respectively) (Fig. 2). Not only does this provide further evidence that *Ppt1*^*−/−*^ astrocytes respond differently to stimulation than their WT counterparts, but is also in marked contrast to data obtained from *Cln3*^*−/−*^ astrocytes which exhibit reduced release of soluble factors following stimulation [35]. Furthermore, lactate secretion from *Ppt1*^*−/−*^ astrocytes was significantly increased 1.4 fold (*p* < 0.05, data not shown) under basal conditions, which may point towards the possibility of mitochondrial dysfunction as shown in aging and prematurely aging mice [39], with mitochondrial failure and a shift in transcriptional activities of lactate dehydrogenases resulted in increased brain lactate levels. Mitochondrial dysfunction is closely linked with oxidative stress and has previously been reported in other forms of the NCLs ([34]; Jalanko et al., 2009).

### Ppt1^−/−^ astrocyte survival is impaired in vitro

**Fig. 3:**
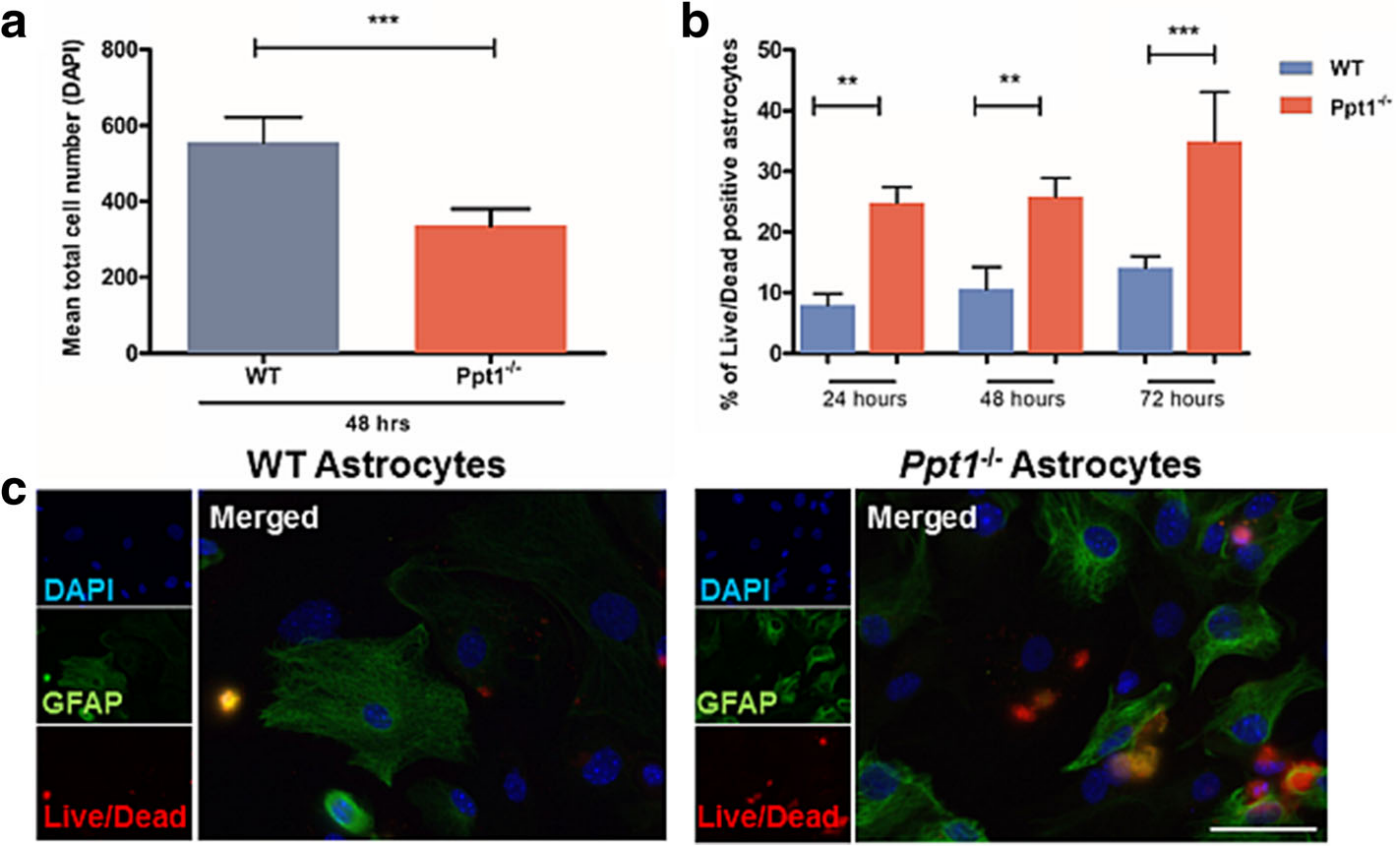
Impaired Survival of Ppt1 deficient (*Ppt1*^*−/−*^) astrocytes. A lower cell number was observed in *Ppt1*^*−/−*^ astrocyte cultures than in wild type (WT) cultures, due to decreased cell survival. **a** Under basal and stimulated conditions the number of *Ppt1*^*−/−*^astrocytes per coverslip was markedly lower than in WT cultures as indicated by significantly reduced counts of cells stained with the nuclear marker DAPI. **b** At 24, 48 and 72 h following seeding onto coverslips cell death was significantly higher in *Ppt1*^*−/−*^ astrocyte cultures than in their WT counterparts. **c** A live/dead cell death marker (red) revealed the increased presence of dying cells in *Ppt1*^*−/−*^astrocyte cultures stained for GFAP (green), compared to those derived from WT mice. (Data shown as Mean ± SEM using a one way ANOVA, n = 3). Scale bar = 50 μm

There are fewer cells that stain positively for GS or S100β in 6 month old *Ppt1*^*−/−*^ mice [29], suggesting that astrocyte survival may be impaired in CLN1 disease in the later stages of disease progression. These data prompted us to investigate *Ppt1*^*−/−*^ astrocyte survival in our cultures, and during our immunofluorescence staining experiments, we noticed fewer DAPI-stained nuclei in *Ppt1*^*−/−*^ astrocyte cultures under all basal and stimulated conditions. Counts of the number of DAPI-stained cells in these *Ppt1*^*−/−*^ astrocyte cultures confirmed they were significantly reduced in number (Fig. 3a). However, no significant differences in the percentage of proliferating cells was observed in *Ppt1*^*−/−*^ astrocyte cultures (data not shown), and as such could not account for significantly reduced number of astrocytes in these cultures. Consequently, a Live/Dead kit was used to investigate cell survival 24, 48 and 72 h after plating the cells onto coverslips (Fig. 3b, c). Across all time points, significantly more astrocytes were positive for both GFAP and for the Live/Dead marker in *Ppt1*^*−/−*^ cultures, indicative of them undergoing cell death (24.83 ± 1.49%; 25.87 ± 1.78%; 34.99 ± 4.66%, at 24, 48 and 72 h respectively) compared to their WT counterparts (7.95 ± 1.08%; 10.57 ± 2.10%; 14.09 ± 1.08% at 24, 48 and 72 h respectively) (Fig. 3b, c). These data reveal that *Ppt1*^*−/−*^ astrocyte survival is markedly impaired in vitro*,* suggesting an inherent vulnerability of *Ppt1*^*−/−*^ astrocytes rather than any defect in their ability to proliferate. Although these tissue culture data for impaired astrocyte survival are consistent with our previous histological evidence from the *Ppt1*^*−/−*^ brain [29], it remains unclear what the extent and nature of astrocyte death may be in vivo, and it will be important to resolve this issue.

### Ppt1^- /−^astrocytes display abnormal Ca^2+^ signalling with increased cytoplasmic Ca^2+^ levels

De-regulated Ca^2+^ signalling has been implicated in the pathogenesis of several neuro-degenerative disorders, including several lysosomal storage disorders (reviewed in Lloyd-Evans, 2016). After the endoplasmic reticulum (ER), the lysosome represents an important intracellular Ca^2+^ store [6, 26]. Thapsigargin is an inhibitor of the sarco/endoplasmic reticulum ATPase (SERCA) that at high concentrations induces Ca^2+^ release from the ER by blocking uptake and uncovering the ER leak channels (Chandrachud et al., 2015). Nigericin is a H^+^/K^+^ ionophore that will release Ca^2+^ from both lysosomes and mitochondria by collapsing the H^+^ gradient [26]. However, prior pre-treatment with ionomycin results in Ca^2+^ release from all intracellular stores apart from lysosomes, where its action is blocked by the lysosomal glyocaylix, meaning that resultant effects of nigericin on Ca^2+^ release are solely from lysosomal stores [26].

**Fig. 4:**
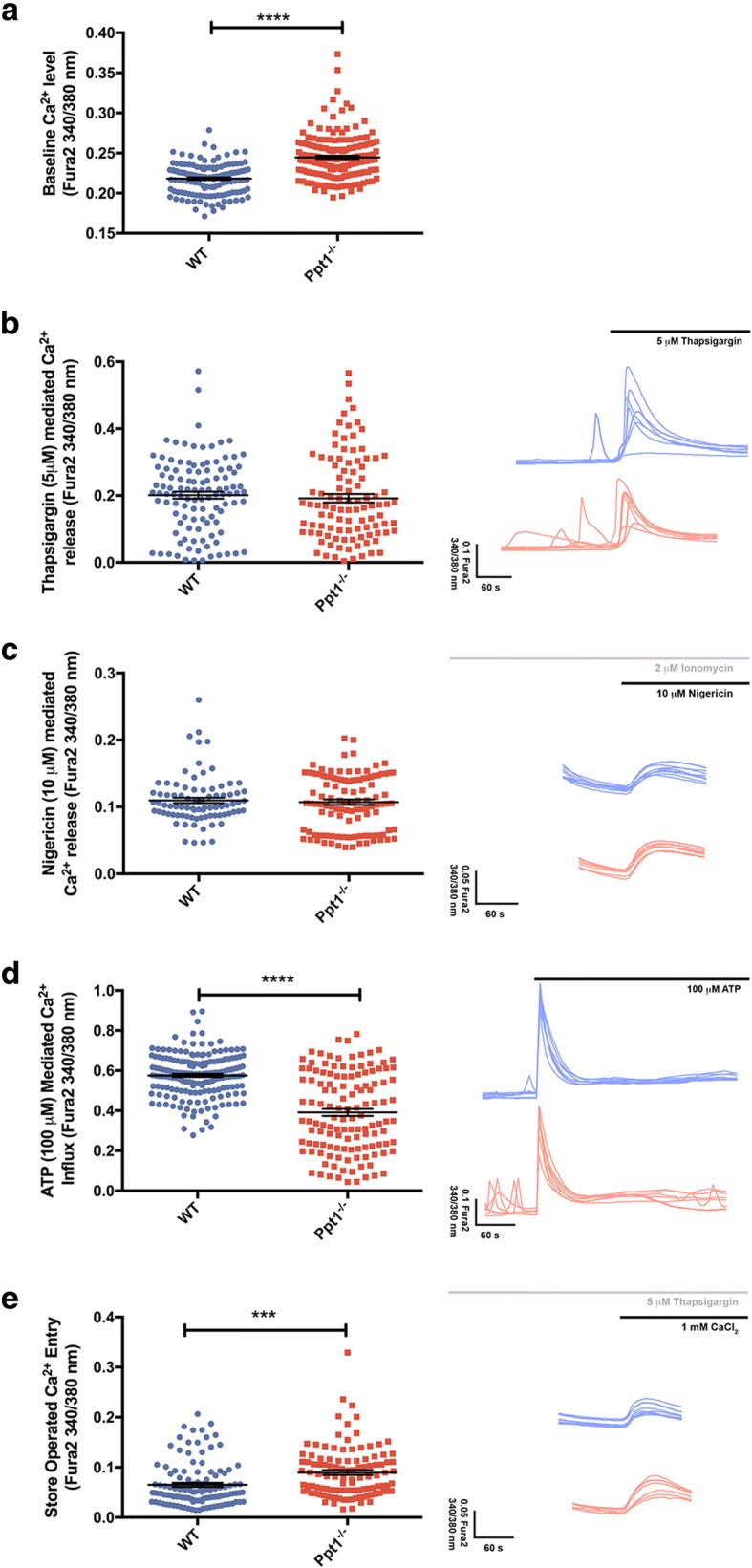
Ppt1 deficient (*Ppt1*^*−/−*^) astrocytes exhibit changes in Ca^2+^ homeostasis. Ca^2+^ measurements were completed 48 h after seeding of wild type (WT) and *Ppt1*^*−/−*^ astrocytes under basal conditions to assess changes in Ca^2+^ homeostasis. Representative Ca^2+^ traces shown for WT (blue) and *Ppt1*^*−/−*^ (red) astrocytes (**b-e**). (**a**) Baseline Ca^2+^ levels measured with Fura2 dye (340/380 nm) were significantly higher in *Ppt1*^*−/−*^astrocytes. (**b**) No statistically significant differences were detected between WT and *Ppt1*^*−/−*^ astrocytes in Ca^2+^ released from endoplasmic reticulum Ca^2+^ stores, mediated by 5 μM thapsigargin. **c** Lysosomal Ca^2+^ release, triggered by 10 μM nigericin was also not significantly altered in *Ppt1*^*−/−*^astrocytes. **d** ATP (100 μM) mediated Ca^2+^ influx was significantly lower in *Ppt1*^*−/−*^astrocytes than in their WT counterparts. **e** Store-operated Ca^2+^ entry, triggered by 5 μM thapsigargin followed by 1 mM CaCl_2_, was significantly higher in *Ppt1*^*−/−*^astrocytes than in WT astrocytes

The base-line level of cytoplasmic Ca^2+^ was significantly elevated in *Ppt1*^*- /−*^ astrocytes (Fig. 4a), suggesting that they are more stressed than their WT counterparts, and this likely caused by the significantly higher number of spontaneous Ca^2+^ release events that we observed in the *Ppt1*^*−/−*^ astrocytes (data not shown). Stimulation of *Ppt1*^*- /−*^ and WT astrocytes with thapsigargin revealed no difference between genotypes in the level of Ca^2+^ released from the ER (thapsigargin, Fig. 4b) or from lysosomes (ionomycin/nigericin, Fig. 4c) or, suggesting Ca^2+^ release from these intracellular stores was unaffected in *Ppt1*^*- /−*^ astrocytes. ATP mediated intracellular Ca^2+^ elevation was significantly lower in *Ppt1*^*- /−*^ astrocytes (Fig. 4d), and following thapsigargin depletion of the ER there was a small, but significant, increase in store operated Ca^2+^ entry in *Ppt1*^*- /−*^ astrocytes (Fig. 4e). Taken together these data suggest that *Ppt1*^*- /−*^ astrocytes display marked abnormalities in Ca^2+^ signalling with elevated levels of both spontaneous Ca^2+^ release events and resting basal cytoplasmic Ca^2+^ that may be related to their pronounced survival defect.

### Ppt1^−/−^ microglia exhibit an activated morphological phenotype in vitro

Alongside activation of astrocytes, microglial activation is also evident in *Ppt1*^*−/−*^ mice as early as three months of age with marked up-regulation of CD68 [27]. We first defined the cellular composition of our microglial cultures. One week after plating microglial cultures showed over 99% of DAPI stained cells expressed CD68 (99.1 and 99.8% CD68 + ve in WT and *Ppt1*^−/−^, respectively; with only 0.89 and 0.14% being GFAP+ve). To assess the in vitro properties of *Ppt1*^*−/−*^ microglia, we first assessed their ability to undergo morphological transformation following pharmacological stimulation with LPS, a response that is impaired in Cln3 deficient microglia [35]. Following stimulation with LPS, WT and *Ppt1*^*- /−*^ microglial cultures were stained with CD68 and α-tubulin, and as described previously cells were classified into 3 morphological subcategories: type 1 cells – microglia with extended processes (non-activated); type 2 cells – microglia with retracted processes (partly activated); type 3 cells– rounded cells with a small soma (fully activated) [35].

**Fig. 5:**
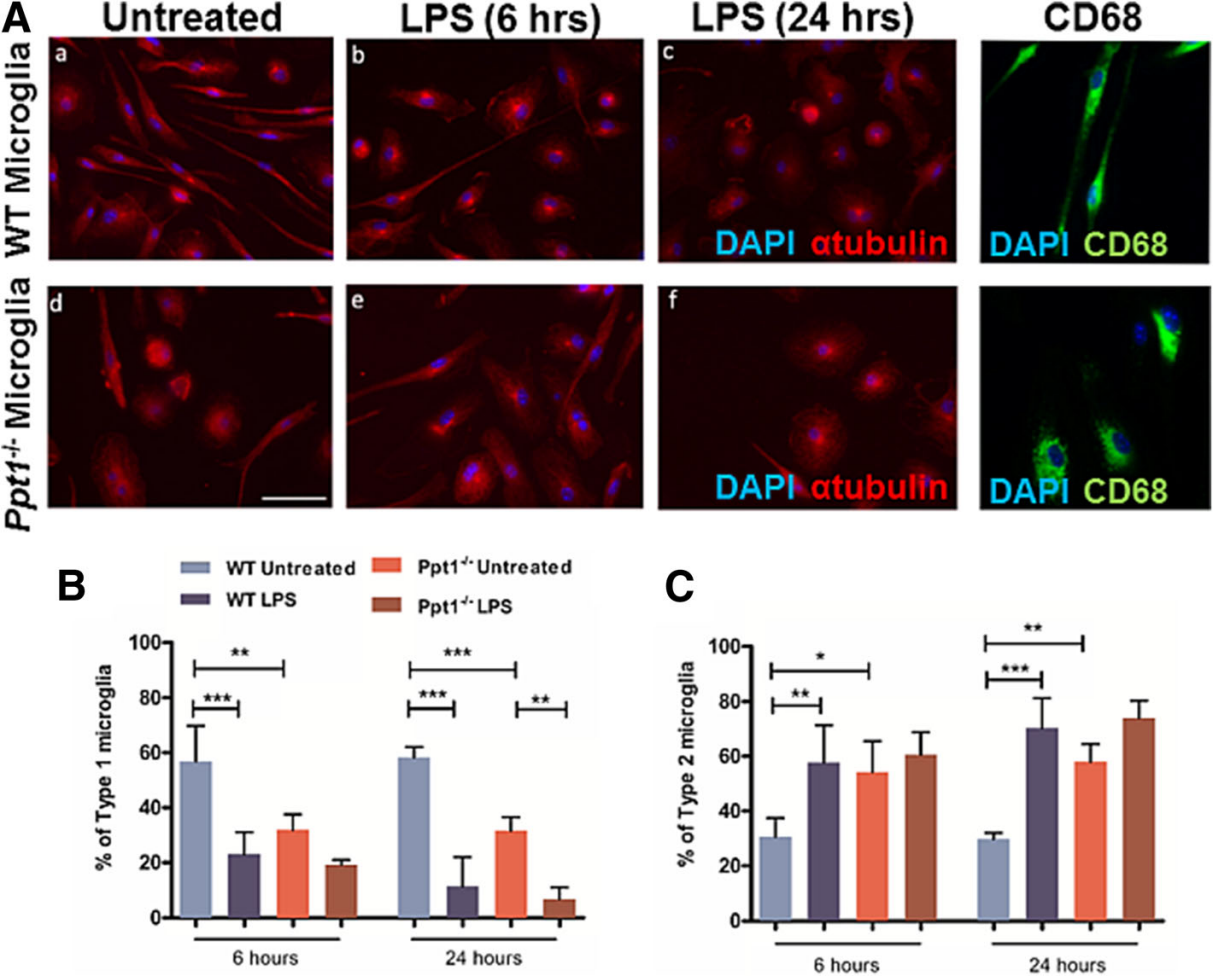
Ppt1 deficient (*Ppt1*^*−/−*^) microglia appear morphologically more activated under basal conditions. To assess microglial activation, we first needed to define their morphology. Type 1 or ‘resting’ microglia exhibit a rod shaped morphology. Type 2 or activated microglia exhibit a round soma and withdrawn processes. Microglial morphology was visualised by staining for CD68 and α-tubulin and 5 random fields were counted per coverslip. α-tubulin was used to assess microglial morphology since CD68 was observed to be more concentrated in the soma and processes were not well visualised using this antibody in these images microglial morphology was visualised with α-tubulin, as CD68 is more concentrated in the soma and processes were not well visualised with this antigen. Morphology was assessed under basal conditions and after stimulation with LPS for 6 and 24 h. **A** *Ppt1*^*−/−*^ microglia appeared morphologically more activated under basal conditions, as more cells with rounded cell bodies were present. Following treatment with LPS, WT microglia assumed a more reactive morphology. Scale bar = 50 μm. **B** A higher percentage of Type 1 microglia were present in WT microglial cultures than in *Ppt1*^*−/−*^microglial cultures, and the percentage of Type 1 microglia was reduced following stimulation. **C** More Type 2 microglia were present under basal conditions in *Ppt1*^*−/−*^ cultures. The percentage of Type 2 microglia increased following stimulation with LPS after 6 and 24 h in WT microglial cultures. (Data shown as Mean ± SEM using a one way ANOVA, *n* = 3)

Under basal conditions, the vast majority of CD68-positive microglia in WT cultures exhibited a bipolar or rod-like morphology (Fig. 5A). Quantitatively these Type 1 cells were the predominant microglial subtype in WT cultures (Fig. 5B). In contrast, far fewer Type 1 cells were present in *Ppt1*^*−/−*^ microglial cultures under basal conditions (Fig. 5B), with the majority of CD68-positive microglia exhibiting a rounded Type 2 morphology (Fig. 5C). As expected, following LPS stimulation WT microglia rounded up, and Type 2 microglia became the prevailing cell type in these cultures (Fig. 5C). Little change in *Ppt1*^*−/−*^ microglial morphology was observed following stimulation for 6 or 24 h, potentially because these mutant microglia already appeared morphologically to be activated (Fig. 5A, C). Very few Type 3 microglia were observed in either WT or *Ppt1*^*−/−*^ microglial cultures (data not shown), consistent with previous observations that prolonged stimulation is required to fully activate microglia to become small and rounded [35]. Taken together these data suggest that *Ppt1*^*−/−*^ microglia appear morphologically to be already activated under basal conditions and do not dramatically alter their morphology any further when stimulated pharmacologically.

### IL-1β secretion is increased in stimulated Ppt1^−/−^ microglia

Oxidative stress has previously been suggested to play a role in poor cellular health in patient derived primary *Ppt1*^*−/−*^ deficient fibroblasts [49]. Because microglia are implicated in mediating the effects of oxidative stress [7, 13], we investigated the release of soluble proteins typically associated with oxidative stress from *Ppt1*^*−/−*^ microglia. ELISA assays of these proteins revealed no significant differences in the release of TNFα, TGFβ, IL-1α, IL-6, IL-10, IL-12 or MCP-1 from WT and *Ppt1*^*−/−*^ microglial cultures (Additional file 1: Figure S1). However, the release of IL-1β was significantly higher in *Ppt1*^*−/−*^ cultures (5.48 ± 4.32%) following 24 h stimulation than in WT cultures (− 8.41 ± 2.04%), suggesting that *Ppt1*^*−/−*^ microglia may adapt a more inflammatory phenotype upon stimulation than WT microglia (Additional file 1: Figure S1). It will be important to provide *Ppt1*^*−/−*^ microglia with an oxidative stress stimulus and determine if the expression of these factors is further altered.

### Ppt1^−/−^ neurons display impaired survival of interneurons and morphological defects

**Fig. 6:**
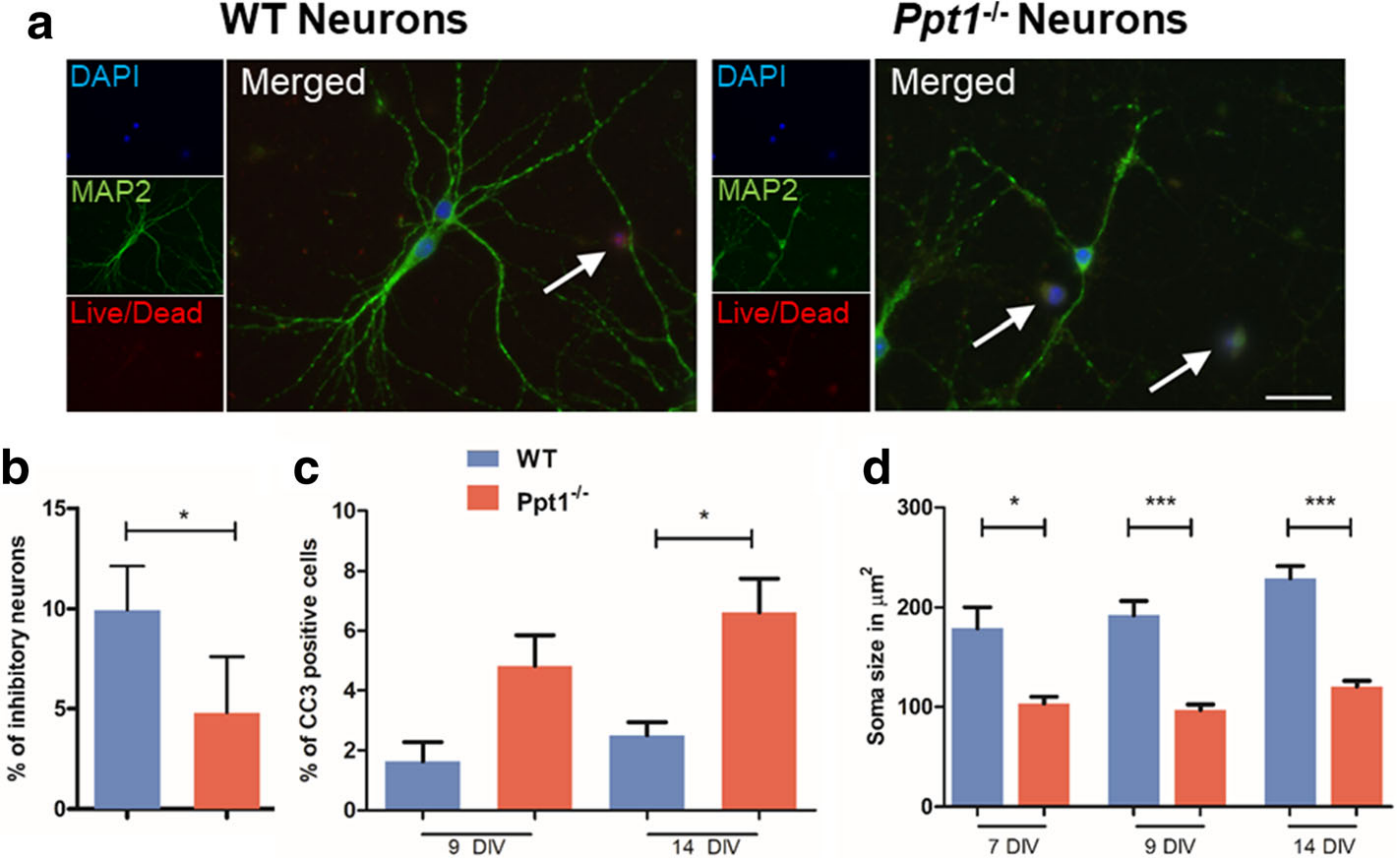
Ppt1 deficient *(Ppt1*^*−/−*^) neuronal cultures exhibit altered culture composition and impaired survival. **a** To analyse neuronal cell death and visualise neuronal morphology, WT and *Ppt1*^*−/−*^neuronal cultures were stained for Map2 and CC3. Pictures of 14 day old cultures stained with nuclear marker DAPI (blue), showing more Map2 positive cells (green) in *Ppt1*^*−/−*^neuronal cultures labelled with CC3 (red) that in WT cultures. Arrows indicate CC3/ DAPI positive cells in WT cultures and Map2/CC3/DAPI positive cells in *Ppt1*^*−/−*^ neuronal cultures. Scale bar = 50 μm. **b** Significantly fewer neurons were positively stained for parvalbumin/calretinin/calbindin in *Ppt1*^*−/−*^ neuronal cultures, indicating that fewer inhibitory neurons are present than in WT neuronal cultures after 7 days in vitro. **c** The percentage of CC3 positive cells did not differ between WT and *Ppt1*^*−/−*^neuronal cultures after 9DIV, but was significantly higher in *Ppt1*^*−/−*^cultures after 14DIV. **d** *Ppt1*^*−/−*^neurons stained for Map2 had significantly smaller soma sizes after 7, 9 and 14 days in vitro (DIV) compared to WT neurons. (Data shown as Mean ± SEM using a one way ANOVA, *n* = 3)

Before defining the impact of Ppt1 deficient astrocytes or microglia upon neurons, we first assessed neuronal culture composition. In neuronal cultures > 82% of cells stained positively for Map2 (16% GFAP positive, 0.3% CD68 positive) after 7 days, and > 93% Map2 positive (5% GFAP positive, 3% CD68 positive) after 14 days. We then compared the survival and morphology of cultured primary cortical neurons derived from WT and *Ppt1*^*−/−*^ mice (Fig. 6a).

Inhibitory neurons are amongst the most vulnerable cells in *Ppt1*^*−/−*^ mice [3], and although there was little difference in overall cell number between *Ppt1*^*−/−*^ and WT neuronal cultures after 7 DIV (data not shown), the percentage of inhibitory neurons stained with a cocktail of antibodies against the interneuron markers parvalbumin, calbindin and calretinin was significantly lower in *Ppt1*^*−/−*^ cortical neuron cultures (4.79 ± 1.41% vs 9.94 ± 1.10% WT) (Fig. 6b). Subsequently, after 14 DIV a significantly higher proportion of cells in *Ppt1*^*−/−*^ neuronal cultures stained positive for cleaved caspase 3 (6.61 ± 1.13% vs 2.49 ± 0.45% WT), suggesting their impaired longer-term survival in vitro (Fig. 6c). We also quantified neuronal morphology as an indicator of in vitro development and neuronal health, and first revealed that the soma size of Map2-positive neurons was significantly smaller in *Ppt1*^*−/−*^ cultures at all time points (Fig. 6d).

**Fig. 7:**
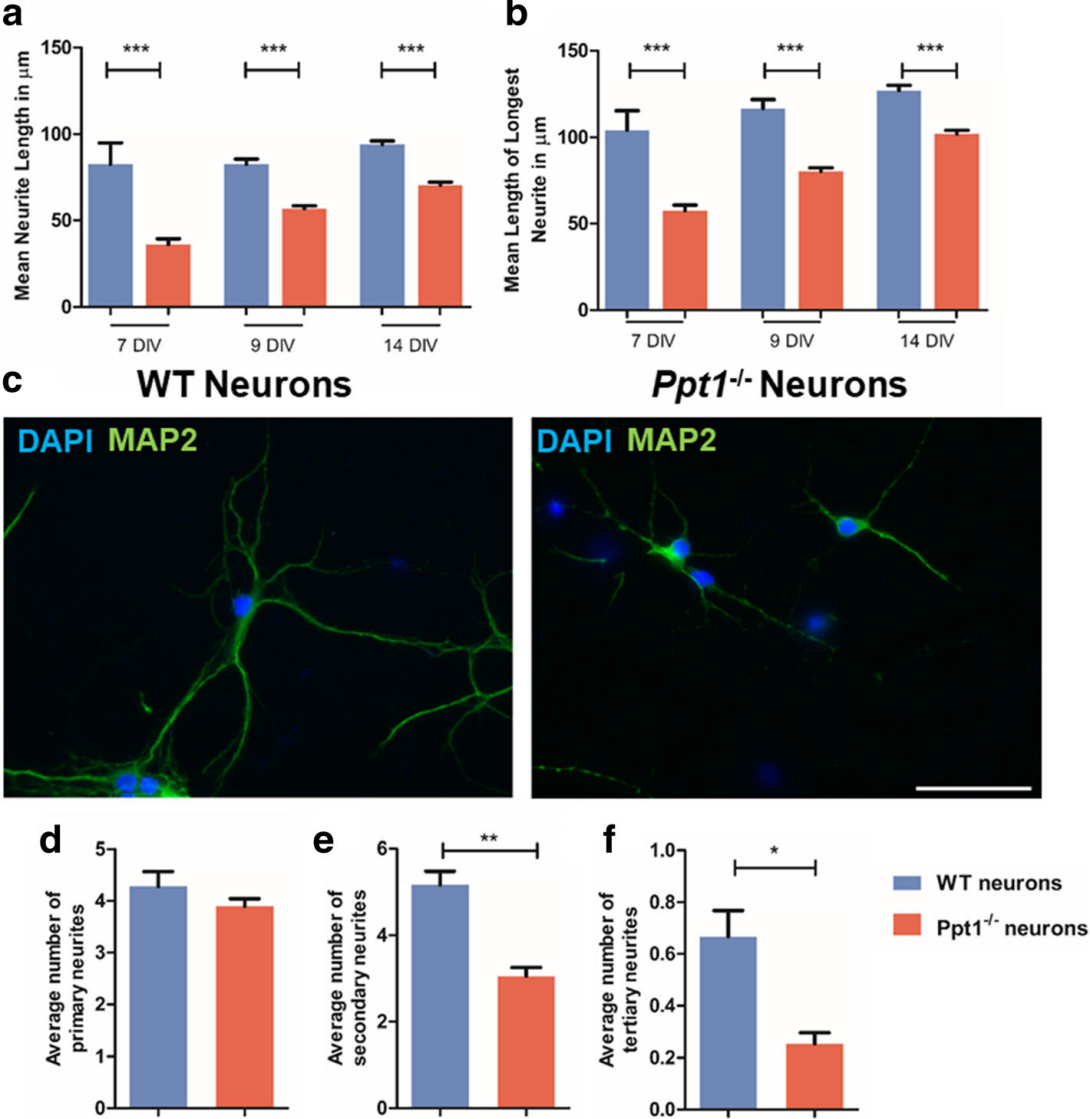
Ppt1 deficient (*Ppt1*^*−/−*^*)* neurite outgrowth is impaired. Wild type (WT) and *Ppt1*^*−/−*^ neuronal cultures were stained with MAP2 to visualise neuronal processes and DAPI to label all nuclei. Neurite length was measured using ImageJ. **a** Mean neurite length is significantly shorter in *Ppt1*^*−/−*^ neuronal cultures than in WT cultures after 7, 9 and 14 days in vitro (DIV). **b** The length of the longest neurites, assumed to be the axon is significantly reduced in *Ppt1*^*−/−*^neurons compared to WT neurons after 7, 9 and 14 DIV. **c** *Ppt1*^*−/−*^ neurons stained for Map2 appear to have shorter neurites than their WT counterparts and appear less complex. **d** The number of primary neurites did not differ between WT and *Ppt1*^*−/−*^ neuronal cultures after 7 days in vitro (DIV). **e** *Ppt1*^*−/−*^ neurons had significantly fewer secondary neurites than their WT counterparts after 7 DIV. **f** Significantly fewer tertiary neurites were present in *Ppt1*^*−/−*^neuronal cultures. (Data shown as Mean ± SEM using a one way ANOVA, n = 3). Scale bar = 50 μm

Assessing neurite complexity (Fig. 7), revealed that at 7, 9, and 14 DIV the mean neurite length was significantly shorter in *Ppt1*^*−/−*^ vs. WT neurons (Fig. 7a), as was the length of their longest neurite, (Fig. 7b) which by 14 DIV is typically the axon [12, 14, 46]. Changes in neurite morphology were readily apparent in Map2 stained cultures (Fig. 7c), but quantitative analysis revealed specific arborization defects. Although the average number of primary neurites was similar between neurons of both genotypes (Fig. 7d), *Ppt1*^*−/−*^ neurons had significantly fewer secondary (Fig. 7e) or tertiary (Fig. 7f) neurites at 7 DIV. Taken together these data suggest that *Ppt1*^*−/−*^ neurons not only show impaired morphology in vitro, suggesting they are in poor health, but also display a moderate impairment in their survival. It will now be important to study neuronal arborization in vivo, to see whether these findings are corroborated in Ppt1-deficient mice.

### Co-cultures reveal the impact of Ppt1 deficiency on cellular interactions

To assess the impact of *Ppt1*^*−/−*^ astrocytes or microglia on each other, or WT and *Ppt1*^*−/−*^ neurons, we grew these cell types together in different combinations (e.g. astrocytes with microglia, neurons with astrocytes, neurons with microglia, neurons with both astrocytes and microglia), using the morphological phenotypes defined above and survival as outcome measures as outcome measures. As such, for co-cultures were stained with Map2 and CC3, soma size as well as neurite length and complexity were measured and the percentage of cells undergoing apoptosis was determined. Where appropriate, microglia were labelled with CD68 and astrocytes with GFAP.

### Detrimental impact of Ppt1^−/−^ astrocytes upon neuron morphology

**Fig. 8:**
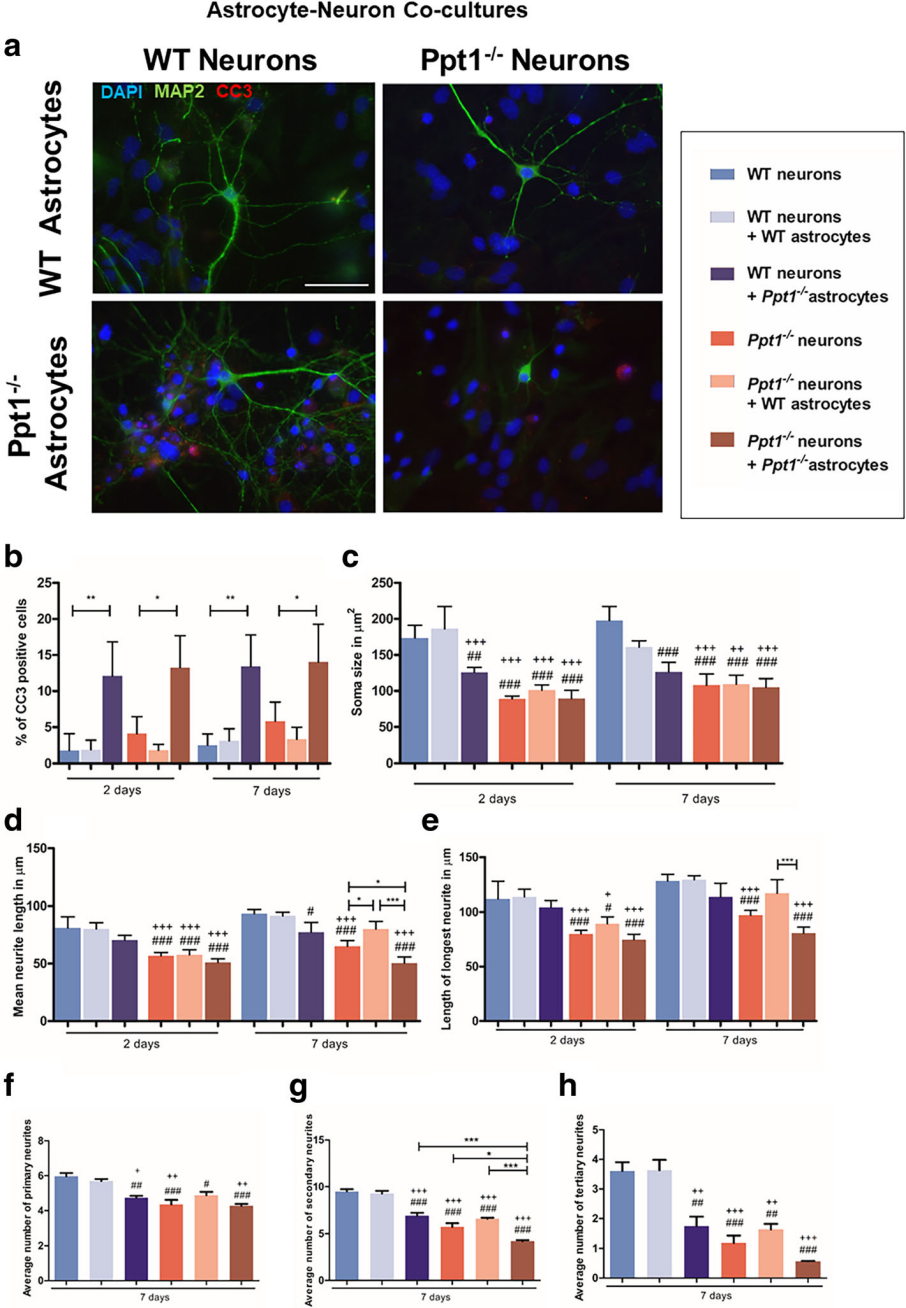
Wild Type (WT) astrocytes ameliorate morphological defects in Ppt1 deficient (*Ppt1*^*−/−*^) neurons. **a** WT and *Ppt1*^*−/−*^ astrocytes and neurons were cultured together for 2 or 7 days, and stained with MAP2 (green) and cleaved caspase 3 (CC3, red) to examine cell survival and neuronal morphology. **b** After both 2 and 7 days in culture, the percentage of CC3 expressing cells was significantly higher in both WT and *Ppt1*^*−/−*^ co-cultures when grown with *Ppt1*^*−/−*^ astrocytes. **c** After both 2 and 7 days in culture, soma size in all *Ppt1*^*−/−*^ culture conditions was significantly smaller than in WT monocultures, and WT neuron/WT astrocyte co-cultures. Although *Ppt1*^*−/−*^ astrocytes had little effect upon *Ppt1*^*−/−*^ neuronal soma size, WT neuron soma size was significantly reduced when grown with Ppt1−/−astrocytes after 2 and 7 days in culture. **d** After 2 days in co-culture, the mean neurite length was shorter in *Ppt1*^*−/−*^ neurons under all conditions. Following 7 days in co-culture, *Ppt1*^*−/−*^ astrocytes had a detrimental impact on both WT and *Ppt1*^*−/−*^ neurite outgrowth, whereas WT astrocytes improved neurite outgrowth in *Ppt1*^*−/−*^ neurons. **e** Axon length in all *Ppt1*^*−/−*^ cultures was significantly shorter than in WT neuron and WT neuron/WT astrocyte co-cultures after 2 days. After 7 days, *Ppt1*^*−/−*^ axon length was significantly reduced in the presence of *Ppt1*^*−/−*^ astrocytes, whereas WT astrocytes significantly improved axon growth. **f** The number of primary neurites was significantly lower in in all *Ppt1*^*−/−*^ cultures as well as WT neuron/ *Ppt1*^*−/−*^ astrocyte cultures. **g** Secondary neurite number was significantly reduced in WT neuron/ *Ppt1*^*−/−*^ astrocyte cultures, *Ppt1*^*−/−*^ neuron cultures *Ppt1*^*−/−*^ neuron/WT astrocyte and *Ppt1*^*−/−*^ neuron/ *Ppt1*^*−/−*^ astrocyte co-cultures compared to WT neuron cultures and WT neuron/WT astrocyte cultures. Secondary neurite number remained significantly lower in *Ppt1*^*−/−*^ neuron/ *Ppt1*^*−/−*^ astrocyte cultures than in WT neuron/ *Ppt1*^*−/−*^ astrocyte cultures, *Ppt1*^*−/−*^ astrocyte cultures and *Ppt1*^*−/−*^ neuron/ *Ppt1*^*−/−*^ astrocyte cultures. **h** The number of tertiary neurites was significantly lower in WT neuron/ *Ppt1*^*−/−*^ astrocyte cultures, *Ppt1*^*−/−*^ neuron cultures, *Ppt1*^*−/−*^ neuron/WT astrocyte and *Ppt1*^*−/−*^ neuron/ *Ppt1*^*−/−*^astrocyte co cultures than in WT neuronal cultures and WT neuron/WT astrocyte cultures. (Data shown as Mean ± SEM using a one-way ANOVA, *n* = 3, # represents significant difference to WT neuron cultures, + represents significant difference to WT neuron/WT astrocyte cultures)

We first assessed different co-culture combinations of astrocytes and neurons of different genotypes, which revealed obvious effects upon neuron survival and morphology (Fig. 8a). Quantifying these changes, cell death in neuron-astrocyte co-cultures, as revealed by CC3 immunostaining, was significantly greater when *Ppt1*^*−/−*^ astrocytes were present (Fig. 8b), either in combination with WT neurons (13.41 ± 2.18%) or *Ppt1*^*−/−*^ neurons (14.03 ± 2.61%). This cell death was predominantly of astrocytes rather than neurons, as there was little correlation between the overall percentage of CC3-positive cells, and those positive for both CC3 and the neuron marker Map2 (data not shown). Less cell death was evident in *Ppt1*^*−/−*^ neuron/WT astrocyte co-cultures (Fig. 8b), but this difference was not statistically significant.

In contrast, *Ppt1*^*−/−*^ astrocytes appeared to have a detrimental impact on neuronal health, as judged by neuronal morphology. When grown with *Ppt1*^*−/−*^ astrocytes, WT neuronal soma size was significantly reduced (Fig. 8c), with decreased mean neurite length (Fig. 8d), but not of the longest neurite, probably the axon (Fig. 8e). These effects were more pronounced after 7 days in co-culture, when WT neurons started resembling *Ppt1*^*−/−*^ neurons morphologically (Fig. 8a). Although *Ppt1*^*−/−*^ astrocytes did not adversely impact *Ppt1*^*−/−*^ neuron soma size after 7 days in co-culture (Fig. 8c), the neurites of these *Ppt1*^*−/−*^ neurons were not only significantly shorter than in *Ppt1*^*−/−*^ neuronal cultures (50.41 ± 2.71 μm vs 65.02 ± 2.51 μm) (Fig. 8d, e), but also had fewer secondary neurites (4.2 ± 0.11vs 5.69 ± 0.4 secondary neurites) (Fig. 8g). In contrast, WT astrocytes appeared to improve *Ppt1*^*−/−*^ neurite outgrowth (80.02 ± 3.31 μm) (Fig. 8d-e), but had no discernible impact on *Ppt1*^*−/−*^ neurite complexity (Fig. 8f-h).

The overall impact of *Ppt1*^*−/−*^ astrocytes appeared to be primarily detrimental to neuronal health, adversely affecting their morphology rather than triggering their death. Although the morphological defects of *Ppt1*^*−/−*^ neurons can be partially restored by WT astrocytes, their survival is not improved, which suggests an intrinsic defect in *Ppt1*^*−/−*^ neuronal survival, despite any possible cross-correction effect of Ppt1 enzyme secreted from WT astrocytes.

### Detrimental impact of Ppt1^−/−^ microglia upon neuron survival

**Fig. 9:**
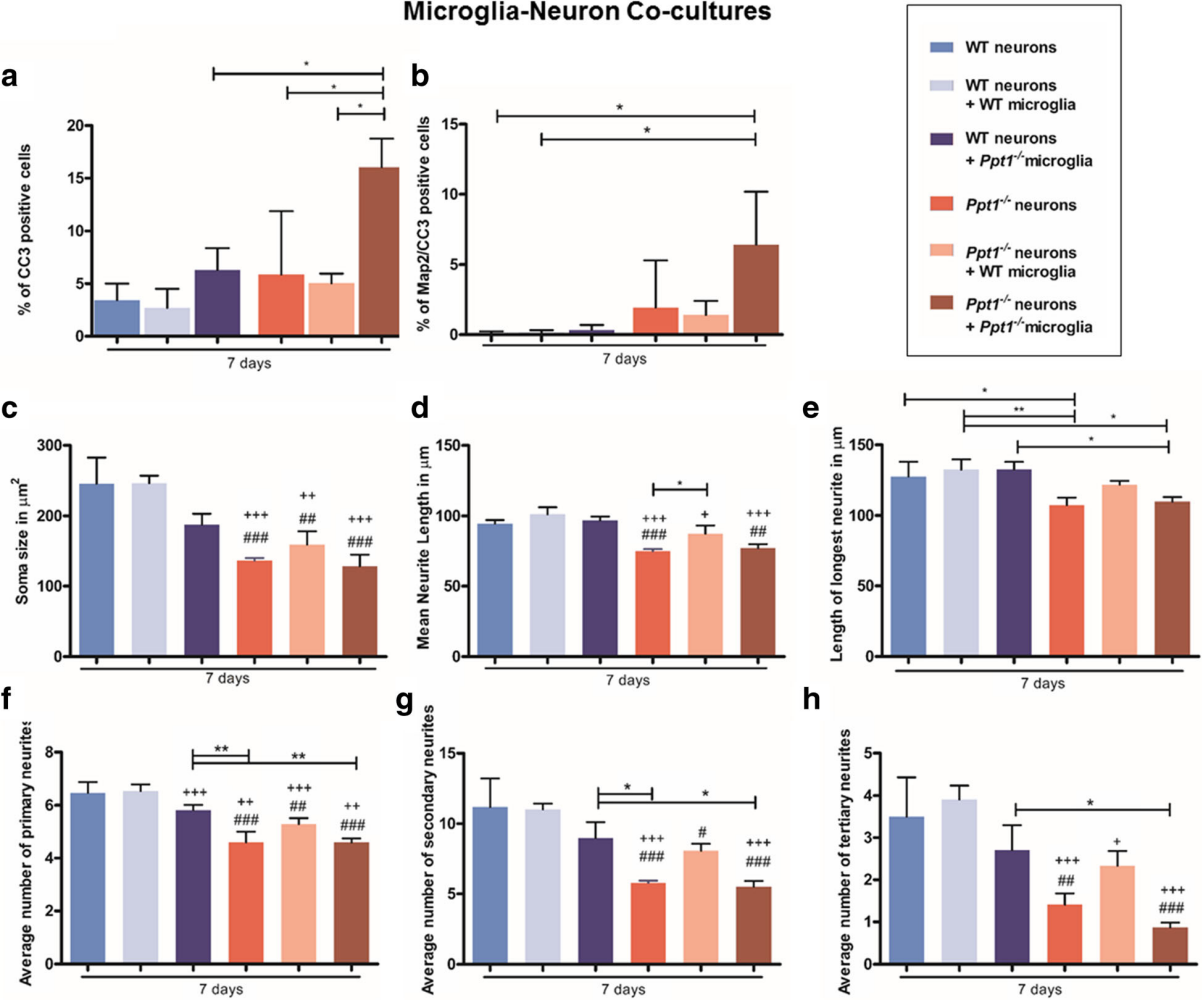
Ppt1 deficient (*Ppt1*^*−/−*^) microglia trigger *Ppt1*^*−/−*^ neuronal death. Wild type (WT) and *Ppt1*^*−/−*^ neurons and microglia were cultured together 7 days, and stained with MAP2 and cleaved caspase 3 (CC3) to examine cell survival and neuronal morphology. **a** The percentage of CC3 expressing cells was statistically significantly higher in *Ppt1*^*−/−*^neuron/ *Ppt1*^*−/−*^ microglial co-cultures than in all other cultures. In contrast *Ppt1*^*−/−*^microglia had a smaller impact on WT neuronal survival. **b** The percentage of Map2/CC3 expressing cells was highly variable within all cultures, although was significantly higher in *Ppt1*^*−/−*^ neuron/ *Ppt1*^*−/−*^microglial co-cultures than in WT neuron and WT neuron/WT microglial cultures. **c** Neuronal soma size was significantly smaller for *Ppt1*^*−/−*^ neurons across all co-cultures, compared to the soma size of WT neurons in either WT monocultures or WT neuron/WT microglial co-cultures. Neuronal soma size was somewhat reduced in WT neuron/ *Ppt1*^*−/−*^ microglial co-cultures, but this difference was not statistically significant. **d** *Ppt1*^*−/−*^ microglia do not have a detrimental effect on *Ppt1*^*−/−*^or WT mean neurite length. However, the presence of WT microglia had a beneficial impact in increasing *Ppt1*^*−/−*^neurite length. **e** Axon length in WT or *Ppt1*^*−/−*^ cultures was unaffected by *Ppt1*^*−/−*^microglia, and the apparently beneficial impact of WT microglia upon axon length was not as pronounced as on mean neurite length. **f** The number of primary neurites was significantly lower across all *Ppt1*^*−/−*^ cultures. **g** Secondary neurite number was significantly reduced in *Ppt1*^*−/−*^neuron cultures, *Ppt1*^*−/−*^ neuron/WT microglia and *Ppt1*^*−/−*^ neuron/ *Ppt1*^*−/−*^ microglia co-cultures compared to WT neuron cultures and WT neuron/WT microglial cultures. A trend towards increased secondary neurite number was observed in *Ppt1*^*−/−*^ neuron/WT microglial cultures. **h** The number of tertiary neurites was significantly lower in *Ppt1*^*−/−*^ neuron cultures, *Ppt1*^*−/−*^neuron/WT microglial and *Ppt1*^*−/−*^ neuron/ *Ppt1*^*−/−*^microglial cultures than in WT neuronal cultures and WT neuron/WT microglial cultures. (Data shown as Mean ± SEM using a one way ANOVA, n = 3; # represents significant difference to WT neuron cultures, + represents significant difference to WT neuron/WT microglia cultures)

We next grew co-cultures of microglia with neurons, in order to assess the impact of these cell types from mice of different genotypes upon each other and revealed a profound impact of *Ppt1*^*−/−*^ microglia upon neuron survival (Fig.9a). Cell death as indicated by CC3 staining appeared significantly higher in *Ppt1*^*−/−*^ neuron/ *Ppt1*^*−/−*^ microglial co-cultures than in WT neuron/ *Ppt1*^*−/−*^ microglial co-cultures, *Ppt1*^*−/−*^ neuronal cultures and *Ppt1*^*−/−*^ neuron/ *WT* microglial co-cultures (Fig. 9a) There were significantly more double- labelled CC3- and Map2-positive cells in *Ppt1*^*−/−*^ neuron/ *Ppt1*^*−/−*^ microglial co-cultures (16.05 ± 1.57%) than in WT neuron/ *Ppt1*^*−/−*^ microglial cultures (6.31 ± 1.19%) (Fig. 9b). While these data suggest a direct cytotoxic effect of *Ppt1*^*−/−*^ microglia upon neurons, a combined effect of *Ppt1*^*−/−*^ microglia with the small number of astrocytes also present in these cultures cannot be ruled out.

We next investigated neuronal morphology in these co-cultures and saw no significant effects of either WT or *Ppt1*^*−/−*^ microglia upon the soma size of neurons of either genotype (Fig. 9c). No detrimental effects of *Ppt1*^*−/−*^ microglia could be observed on neurite outgrowth (Fig. 9d-e), or neurite complexity (Fig. 9f-h). However, in *Ppt1*^*−/−*^ neuron/WT microglia co-cultures there were more secondary (8.1 ± 0.29 vs 5.8 ± 0.08 *Ppt1*^*−/−*^ neurons) and tertiary neurites (2.33 ± 0.2 vs 1.41 ± 0.15 *Ppt1*^*−/−*^ neurons) (Fig. 9g-h) than in *Ppt1*^*−/−*^ neuron cultures, and these neurites were longer (74.96 ± 0.84 μm *Ppt1*^*−/−*^ neuron vs. 87.37 ± 3.35 μm *Ppt1*^*−/−*^*/* WT co-culture) (Fig. 9d-e), suggesting a positive influence of WT microglia upon neurite outgrowth and complexity, perhaps by their secretion of Ppt1 enzyme.

Overall, these data reveal that, compared to *Ppt1*^*−/−*^ astrocytes, *Ppt1*^*−/−*^ microglia exert less of an influence upon neuronal morphology, but exert a significant negative effect upon the survival of *Ppt1*^*−/−*^ neurons with much less of an impact upon WT neurons. As such, *Ppt1*^*−/−*^ microglia do not appear to be intrinsically neurotoxic, but it appears that *Ppt1*^*−/−*^ neurons may simply be more vulnerable to their influence.

### Astrocytes drive microglial activation in Ppt1^−/−^ mixed glial cultures

**Fig. 10:**
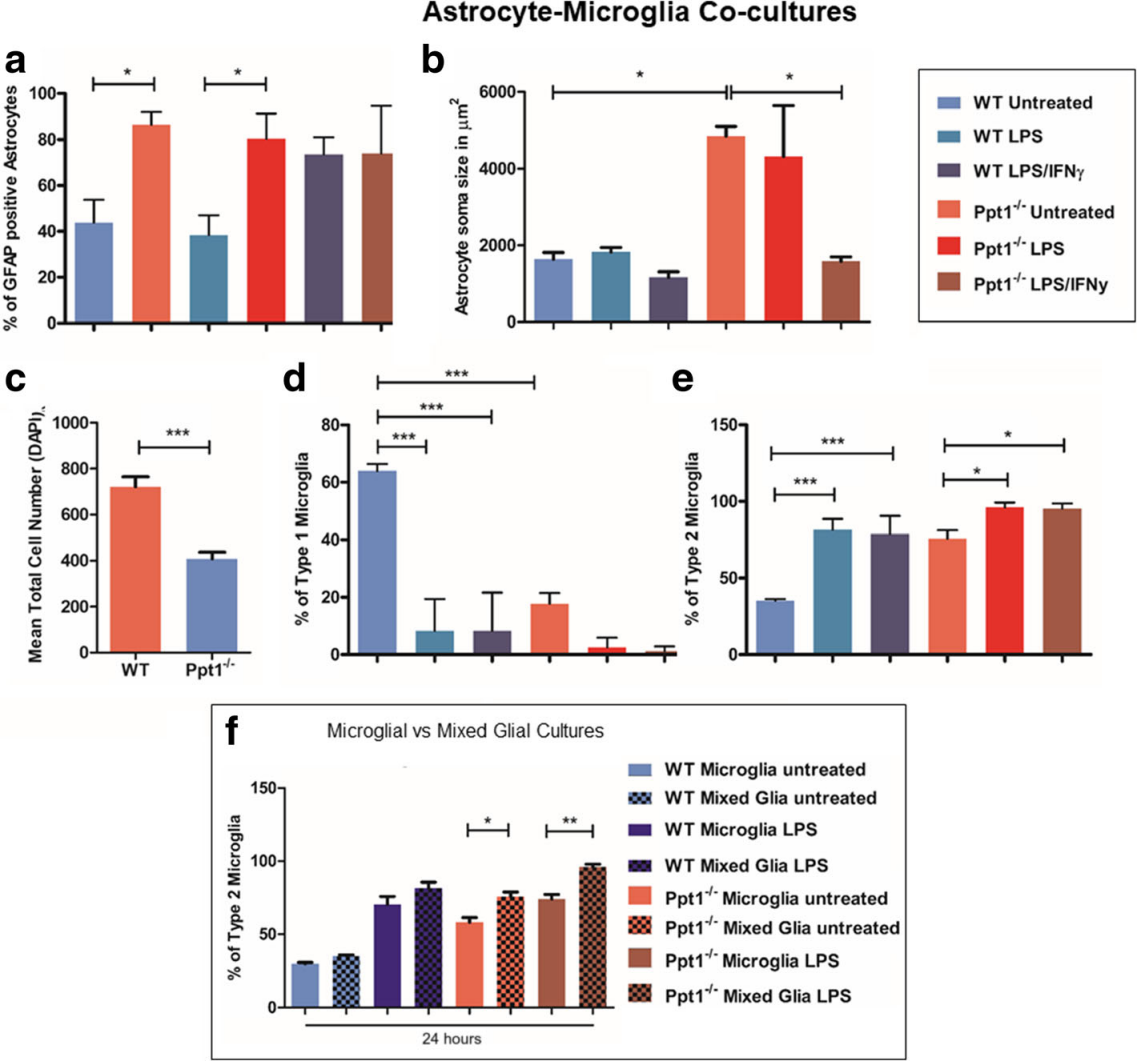
Ppt1 deficient (*Ppt1*^*−/−*^) astrocytes drive microglial activation. *Ppt1*^*−/−*^ and wild type (WT) cultures were maintained as mixed glial (astrocyte and microglial) cultures to examine whether monoculture phenotypes were maintained under basal and stimulated (LPS or LPS/IFNγ) conditions. **a** Under basal conditions, the proportion of cells expressing the astrocyte marker glial fibrillary acidic protein (GFAP) expression was higher in *Ppt1*^*−/−*^ mixed glial cultures than in their WT counterparts. GFAP expression did not increase in WT or *Ppt1*^*−/−*^ mixed glial cultures following addition of LPS, but did increase in WT following stimulation with LPS/IFNγ. **b** Under basal conditions, compared to WT cultures, *Ppt1*^*−/−*^ astrocytes exhibited a larger cell body size, which was decreased dramatically in response to exposure to LPS/IFNγ but not LPS only. **c** The mean total cell number as indicated by DAPI staining was statistically significantly lower in *Ppt1*^*−/−*^ mixed glial cultures. **d** The percentage of Type 1 (resting, elongated) microglia was significantly higher in WT cultures under basal conditions than in *Ppt1*^*−/−*^ mixed glial cultures. **e** The percentage of Type 2 (activated, rounded) microglia was higher in *Ppt1*^*−/−*^ mixed glial cultures under basal conditions. In both WT and *Ppt1*^*−/−*^ mixed glial cultures the percentage of Type 2 microglia was increased following stimulation with LPS, or with LPS/IFNγ. **f** *Ppt1*^*−/−*^ microglia appear morphologically more activated in mixed glia cultures than in microglial cultures, as indicated by a significantly higher percentage of Type 2 microglia under both basal and stimulated (LPS) conditions in mixed glial cultures. (Data shown as Mean ± SEM using a one way ANOVA, n = 3)

Mixed glial cultures containing both astrocytes and microglia of either genotype were grown to examine whether the presence of other glial cells may improve or exacerbate the phenotypes exhibited by either cell type in monocultures. The presence of microglia appeared to have little impact on *Ppt1*^*−/−*^ astrocytes, as the percentage of GFAP-expressing cells remained significantly higher in *Ppt1*^*−/−*^ cultures under basal conditions and after exposure to LPS alone than in WT cultures (Fig. 10a). Similarly, *Ppt1*^−/−^ astrocyte soma size in these mixed glial cultures was consistently significantly larger than that of WT astrocytes, until stimulated with LPS and IFNγ, after which *Ppt1*^−/−^ astrocyte cell body size decreased dramatically (Fig. 10b), as we had seen in astrocyte monocultures (Fig. 1b). Significantly fewer cell nuclei were counted in *Ppt1*^−/−^ mixed glial cultures (408.1 ± 28.89 vs 720.7 ± 43.19 WT) (Fig. 10c), suggesting that the presence of WT microglia does not improve *Ppt1*^−/−^ astrocyte survival.

The phenotypes seen in *Ppt1*^*−/−*^ microglial monocultures also persisted in mixed astrocyte:microglial cultures. Under basal conditions, Type 1 microglia (63.99 ± 1.39%) were  the predominant cell type in WT cultures (Fig. 10d), which transformed into rounded Type 2 activated microglia following stimulation (81.53 ± 4.03% LPS, 78.82 ± 6.74% LPS/IFNγ) (Fig. 10e). Under all conditions, activated Type 2 microglia (75.70 ± 3.17% basal; 96.02 ± 1.84% LPS; 95.31 ± 1.91% LPS/IFNγ) were the prevailing type of microglia present in *Ppt1*^*−/−*^ microglial cultures (Fig. 8e), however the percentage of activated Type 2 microglia was significantly higher in mixed astrocyte:microglial cultures than in microglial monocultures (Fig. 10e). Furthermore, following stimulation with LPS only or LPS and IFNγ, the percentage of Type 2 microglia significantly increased in *Ppt1*^*−/−*^ mixed astrocyte:microglial cultures (Fig. 10f), which was not observed in cultures of microglia alone. These data suggest that *Ppt1*^*−/−*^ astrocytes may drive microglial activation, and also induce a greater microglial response following pharmacological stimulation.

### Neuron/astrocyte/microglial co-cultures

**Fig. 11:**
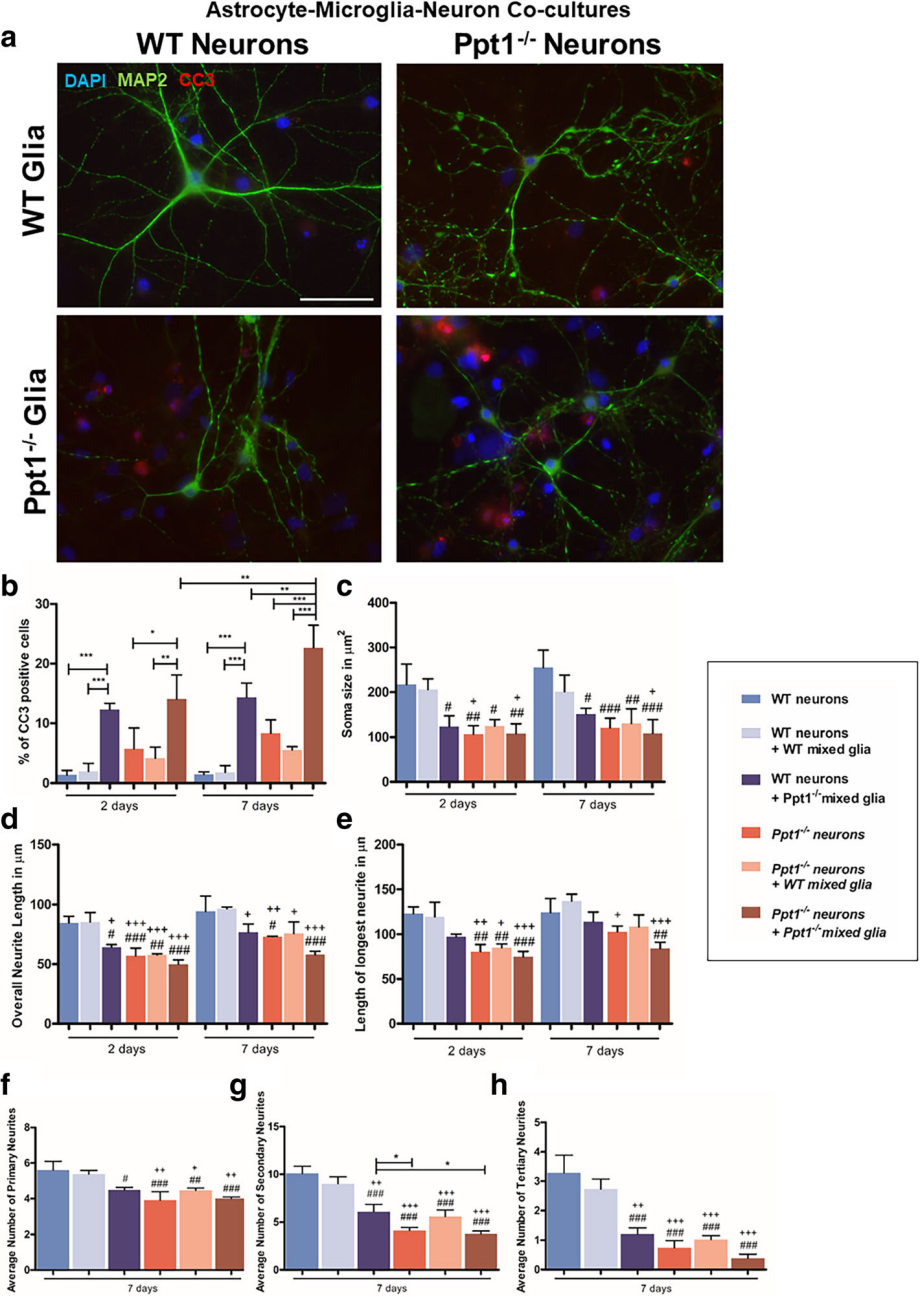
Ppt1 deficient (*Ppt1*^*−/−*^) astrocyte/microglia interactions escalate cell death in *Ppt1*^*−/−*^ neuron/ *Ppt1*^*−/−*^ mixed glial cultures after prolonged co-culturing. **a** Wild type (WT) and *Ppt1*^*−/−*^ neurons and mixed glia (astrocytes and microglia) were cultured together for 2 and 7 days and stained with MAP2 and cleaved caspase 3 (CC3) to examine cell survival and neuronal morphology. Cells were stained with neuronal marker Map2 (green), cell death marker CC3 (red) and nuclear stain DAPI (blue). After 7 days in co-culture, more CC3 positive cells were observed in WT neuron/ *Ppt1*^*−/−*^ mixed glial co-cultures and *Ppt1*^*−/−*^ neuron/ *Ppt1*^*−/−*^ mixed glial co-cultures. Scale bar = 50 μm. **b** The percentage of CC3 positive cells was significantly higher in WT neuron/ *Ppt1*^*−/−*^ mixed glial co-cultures, and *Ppt1*^*−/−*^ neuron/ *Ppt1*^*−/−*^ mixed glial co-cultures after both 2 and 7 days. After 7 days, the percentage of CC3 positive cells had significantly increased within *Ppt1*^*−/−*^ neuron/ *Ppt1*^*−/−*^ mixed glial co-cultures. **c** Soma size across *Ppt1*^*−/−*^ cultures was smaller than in WT and WT neuron/WT mixed glial co-cultures after both 2 and 7 days in culture. WT neuron size was significantly reduced following the addition of *Ppt1*^*−/−*^ mixed glia at both time points. **d** After both 2 and 7 days in culture, mean neurite length was shorter across all *Ppt1*^*−/−*^ cultures than in WT monocultures or WT neuron/WT mixed glial co-cultures. WT neurite length was significantly reduced in the presence of *Ppt1*^*−/−*^ mixed glia after 2 and 7 days in culture. *Ppt1*^*−/−*^ neurite length was also somewhat reduced after 7 days in co-culture with *Ppt1*^*−/−*^ mixed glia. **e** *Ppt1*^*−/−*^ axon length was consistently shorter than that of WT neurons, and although WT axon length was somewhat reduced in WT neuron/ *Ppt1*^*−/−*^ mixed glial co-cultures, this was not statistically significant. **f** The number of primary neurites was significantly lower in WT neuron/ *Ppt1*^*−/−*^ mixed glial cultures, as well as in all *Ppt1*^*−/−*^ cultures. **g** Secondary neurite number was significantly reduced in WT neuron/ *Ppt1*^*−/−*^ mixed glial cultures, *Ppt1*^*−/−*^ neuron cultures, *Ppt1*^*−/−*^ neuron/WT mixed glial and *Ppt1*^*−/−*^ neuron/ *Ppt1*^*−/−*^ mixed glial co-cultures compared to WT neuron cultures and WT neuron/WT mixed glial cultures. Secondary neurite number remained significantly lower in *Ppt1*^*−/−*^ neuron and *Ppt1*^*−/−*^ neuron/*Ppt1*^*−/−*^ mixed glial cultures than in WT neuron/*Ppt1*^*−/−*^ mixed glial cultures. **h** The number of tertiary neurites was significantly lower in WT neuron/*Ppt1*^*−/−*^ mixed glial cultures, *Ppt1*^*−/−*^ neuron cultures, *Ppt1*^*−/−*^ neuron/WT mixed glial and *Ppt1*^*−/−*^ neuron/*Ppt1*^*−/−*^ mixed glial cultures than in WT neuronal cultures and WT neuron/WT mixed glial cultures. (Data shown as Mean ± SEM using a one way ANOVA, n = 3; # represents significant difference to WT neuron cultures, + represents significant difference to WT neuron/WT microglia cultures)

The presence of even some *Ppt1*^*−/−*^ astrocytes appears to influence morphological measures of microglial activation (Fig. 10), and may also potentially influence any negative impact of microglia (Fig. 9). Therefore, we hypothesised that growing mixed cultures that contain proportionately more astrocytes than microglia, a situation that may more closely reflect the composition if the brain in vivo [20], might result in the most detrimental environment for neurons. This appeared to be the case with progressively more CC3-positive cells being observed in these co-cultures with time (Fig. 11), with a corresponding negative influence upon neuronal morphology (Fig. 11a).

After 7 days in co-culture, cell death levels were at their highest in *Ppt1*^*−/−*^ neuron/ *Ppt1*^*−/−*^ mixed glial cultures (22.67 ± 2.17% of cells CC3 + ve) (Fig. 11b), a significant increase compared to cell death after 2 days of co-culturing *Ppt1*^*−/−*^ neurons with *Ppt1*^*−/−*^ mixed glia (14.05 ± 2.33% of cells CC3 + ve), which was comparable to that in WT neuron/ *Ppt1*^*−/−*^ mixed glial co-cultures. In contrast, there appeared to be a moderate protective effect of WT astrocytes resulting in decreased cell death in *Ppt1*^*−/−*^ neuron/WT mixed glial cultures (5.58 ± 0.32% of cells CC3 + ve) (Fig. 11b), perhaps as a result of cross correction by Ppt1 enzyme secreted by these WT cells.

Exploring effects upon neuronal morphology revealed that the soma size of *Ppt1*^*−/−*^ neurons was not affected by the presence of either WT or *Ppt1*^*−/−*^ mixed glia, and remained significantly smaller than their WT counterparts (Fig. 11c). However, when WT neurons were grown with *Ppt1*^*−/−*^ mixed glia, their neuronal soma size was significantly reduced, suggesting a detrimental impact of these Ppt1 deficient astrocytes and microglia upon neuronal health (Fig. 11c). Indeed, *Ppt1*^*−/−*^ astrocytes and microglia also had a pronounced and rapid impact upon WT average neurite length (Fig. 11d-e), which was significantly shorter after only 2 days in co-culture (64.25 ± 1.24 μm vs WT neurons 84.72 ± 3.13 μm). Although some growth in WT neurite length was apparent with continued time in culture, average neurite length remained shorter after 7 days of co-culture in WT neuron/ *Ppt1*^*−/−*^ mixed glial cultures (76.70 ± 4.01 μm) vs. WT/WT cultures (96.59 ± 0.80 μm). The combined presence of *Ppt1*^*−/−*^ astrocytes and microglia also significantly impacted neurite complexity with significant reductions in the average number of primary (4.48 ± 0.09 vs 5.61 ± 0.28 WT neurons, Fig. 11f), secondary (6.07 ± 0.45 vs 10.08 ± 0.44 WT neurons, Fig. 11g) and tertiary neurites in WT neurons (1.21 ± 0.12 vs 3.29 ± 0.35 WT neurons, Fig. 11h). In contrast, the presence of WT astrocytes and microglia produced no significant improvements in *Ppt1*^*−/−*^ neuronal soma size (Fig. 11c), neurite outgrowth (Fig. 11d-e) or complexity (Fig. 11f-h).

Taken together, these data suggest that the presence of both *Ppt1*^*−/−*^ astrocytes and microglia has the most significant detrimental effects upon neurons, not just upon their morphology, but also upon the survival of both WT and *Ppt1*^*−/−*^ neurons. WT astrocytes and microglia were not capable of rescuing the pronounced defects of *Ppt1*^*−/−*^ neurons, providing further evidence that these neurons are intrinsically more vulnerable than WT neurons.

## Discussion

The purpose of undertaking this tissue culture study was not to replicate disease progression in the Ppt1 deficient brain, but rather to assess intrinsic defects in *Ppt1*^*−/−*^ glial biology. Indeed, in addition to detailing defects in the morphology and survival of neurons, this study is the first to characterize *Ppt1*^*−/−*^ glia in vitro*.* Our data reveal that not only do *Ppt1*^*−/−*^ microglia and astrocytes exhibit a range of abnormal phenotypes, they also appear capable of harming neurons, especially if these neurons are Ppt1 deficient. Whilst *Ppt1*^*−/−*^ astrocytes predominantly affect neuronal morphology, the presence of *Ppt1*^*−/−*^ microglia alone was enough to impact neuron survival, a negative influence that was greater if both *Ppt1*^*−/−*^ astrocytes and *Ppt1*^*−/−*^ microglia were present, resulting in pronounced neuron loss. These data suggest that glial cells are more affected by Ppt1 deficiency than previously anticipated, and this may directly influence neuron survival in CLN1 disease and it will be important to explore this also occurs in vivo. However, as discussed below, these defects in Ppt1 deficient glia are quite distinct from those we recently reported in similar experiments in CLN3 disease [35], another member of this group of disorders in which a negative influence of functionally compromised glia upon neuronal survival is evident. Taken together, these data provide further evidence that although these disorders broadly share similar features, they may differ markedly in how individual cell types are impacted by disease.

### Effects of Ppt1 deficiency upon glial biology

Compared to the morphologically attenuated glial activation that is evident in CLN3 disease [35], mouse models of CLN1 disease exhibit much more pronounced and early onset activation of both astrocytes and microglia in the same CNS regions that later display the most neuron loss. This includes the thalamocortical system [23], cerebellum [29], and long before these events occur in the brain this glial activation is also evident at all levels of the spinal cord [43]. These reactive events are accompanied by a pronounced upregulation of chemokines and cytokines in vivo [29], that can be normalized by a combination of brain directed gene therapy and anti-inflammatory drugs [28]. Our data demonstrate that cultured *Ppt1*^*−/−*^ astrocytes are very responsive to pharmacological stimulation (Figs. 1), and *Ppt1*^*−/−*^ microglia appear morphologically more activated even under basal unstimulated conditions (Fig. 5). This is quite different to the properties of cultured *Cln3*^*−/−*^ astrocytes and microglia that both respond slowly and incompletely [35], mirroring the relative extent of morphological transformation of these cell types with disease progression in murine CLN1 and CLN3 diseases in vivo [23, 35, 37, 38]. Another marked phenotypic difference between glia isolated from mouse models of these two forms of NCLs is the reduced secretion of chemokines and cytokines by *Cln3*^*−/−*^ astrocytes and microglia in culture [35], compared to the elevated secretion of a subset of these factors by *Ppt1*^*−/−*^ astrocytes (Fig. 2) and microglia (Additional file 1: Figure S1), data that mirrors in vivo findings for much broader elevation of such factors [29]. Any comparison between in vitro and in vivo studies must be made with caution. However, there are differences between these expression profiles in terms of which chemokines are upregulated. These may reflect the markedly different ages of the mice when these studies were performed, the inherent differences in a tissue culture environment and the living brain, as well as the pharmacological stimulation used in the current in vitro study.

Another fundamental difference between data from Cln1 and Cln3 glia, is that compared to the disruption of intermediate filaments seen in *Cln3*^*−/−*^ astrocytes [35], the current study found no evidence for any similar cytoskeletal defects in *Ppt1*^*−/−*^ astrocytes (data not shown). Taken together, these data emphasize that although glia from both of these mouse models of NCL display a variety of abnormal phenotypes in culture, and that these consistently affect astrocytes more than microglia, the nature and extent of these glial defects differ markedly between CLN1 and CLN3 disease.

One of the most striking findings of the current study is the compromised survival of *Ppt1*^*−/−*^ astrocytes in all culture conditions, a phenotype not exhibited by *Cln3*^*−/−*^ astrocytes in vitro [35]. It will be important to determine if this vulnerability of *Ppt1*^*−/−*^ astrocytes is related to abnormal calcium signalling properties that they display (Fig.4), but it might be expected that these raised cytoplasmic calcium levels would contribute to their death. It will be crucial to determine if *Ppt1*^*−/−*^ astrocyte survival is also impaired in vivo to a similar extent that we have observed in vitro. Although there is already evidence for fewer astrocytes expressing S100β and glutamine synthetase in *Ppt1*^*−/−*^ mice during the later stages of the disease [29], which points towards a loss of these astrocyte populations in vivo, this issue is yet to be fully resolved. Our in vitro findings do provide further corroborative evidence for an inherent vulnerability of *Ppt1*^*−/−*^astrocytes, and this would be expected to adversely impact neurons and their survival during CLN1 disease progression. However, the exact mechanisms underlying such cell-specific vulnerability of astrocytes remain unclear and will need to be investigated in subsequent in vivo studies.

### Effects of Ppt1 deficiency upon neurons

CLN1 disease is perhaps the most profoundly neurodegenerative form of NCL, with near total cortical neuron loss at autopsy [18]. However, the mechanisms by which a deficiency in a de-palmitoylating lysosomal enzyme leads to this remarkably dramatic neuron loss remain unclear. Previous studies culturing neurons derived from *Ppt1*^*−/−*^ mice surprisingly did not reveal any overt defect in their survival, or firing properties, but did find alterations in the synaptic vesicle pool size [47]. These are suggestive of an early stage synaptic pathology, and *Ppt1*^*−/−*^ mice do go on to display progressive changes in synaptic organization within the thalamocortical system [24]. Our data in this study provide new evidence that *Ppt1*^*−/−*^ cortical neurons show compromised survival in our culture conditions (Fig. 6c), display a markedly smaller cell soma (Fig. 6d) and compromised neurite organization in vitro, that involves secondary and tertiary neurites rather than those arising directly from the soma (Fig. 7d-f). This neuron loss was most pronounced for GABAergic interneurons (Fig. 6b), consistent with in vivo data from these *Ppt1*^*−/−*^ mice [3, 23]. Taken together, these data reveal that Ppt1 deficiency does not by itself result in the same profound extent of neuron loss seen in vivo, suggesting that other non-neuron-intrinsic mechanisms may operate to influence neurodegeneration in this disorder.

### Assessing the glial contribution to neuron loss

There is an increasing body of evidence that glial activation and/or dysfunction have an active role in the pathogenesis of several neurodegenerative disorders [1, 36, 40, 44]. Although not necessarily themselves instigators of neurodegeneration, any disruption of the normal support functions served by astrocytes or microglia may plausibly lead to neuron dysfunction or loss. The concept that glial cells can contribute to disease severity and accelerate its progression is perhaps most advanced in ALS and Parkinson disease, but it is becoming apparent this principle also extends to several lysosomal storage disorders. Lysosomal dysfunction and disrupted autophagy were thought to lead to the toxic role of astrocytes in multiple sulfatase deficiency, with the suggestion that a genetic defect in astrocytes alone was sufficient to drive the disease [11]. Microglial activation has also been suggested to contribute adversely to the pathogenesis in various LSDs including MPSI, MPSIIIB, Sandhoff disease and GM2 gangliosidosis [22, 33, 48], but the extent to which this happens in the NCLs is less clear.

The close correlation between the sites where early localized glial activation occurs in every form of NCL, and the extent of subsequent neuron loss [9, 34], has always begged the question of whether these events may be mechanistically related to one another. So far, the role of glia in these disorders has been investigated in most depth in CLN3 disease, and there is recent evidence for a negative influence of Cln3 deficient microglia [51] and astrocytes [4] upon neurons. We have recently extended these observations, using very similar primary culture and co-culture systems used in the present study [35]. This approach revealed a variety of defects in the biology of *Cln3*^*−/−*^ microglia and astrocytes, and when grown in co-cultures, these dysfunctional glia harmed healthy neurons and resulted in the death of *Cln3*^*−/−*^ neurons [35]. These data prompted us to investigate whether there was any influence of *Ppt1*^*−/−*^ astrocytes and microglia upon neuron health in CLN1 disease.

Despite the marked differences in the phenotypes displayed in vitro by *Ppt1*^*−/−*^ glia (this study) and *Cln3*^*−/−*^ glia [35], a consistent feature is that astrocytes and microglia deficient in either gene exert a negative influence of upon neuronal morphology and survival. The relative contributions of either *Cln3*^*−/−*^ astrocytes or microglia to these processes are yet to be defined, as are the underlying mechanisms, but the current study has provided the first insights into the negative influence of glia in CLN1 disease. Our data reveals that this adverse impact varies between cell types, with *Ppt1*^*−/−*^ astrocytes only exerting a moderate influence on neuronal morphology (Fig. 8), whereas *Ppt1*^*−/−*^ microglia are capable of inducing the death of neurons (Fig. 9), especially if these neurons are themselves Ppt1 deficient. These differences likely reflect how each of these cell-types are compromised by Ppt1 deficiency, and it will be important to investigate these mechanisms by transcriptional profiling. Whatever the underlying mechanisms may be, this adverse influence appeared to be exacerbated when both *Ppt1*^*−/−*^ astrocytes and *Ppt1*^*−/−*^ microglia were present (Fig. 11). This is consistent with recent data suggesting that under certain conditions microglia may prime astrocytes to become neurotoxic [25]. However, the specific factors implicated in this priming mechanism were not all upregulated by *Ppt1*^*−/−*^ microglia, and it may be that different mechanisms operate in CLN1 disease. However, data from our co-cultures of *Ppt1*^*−/−*^ astrocytes and *Ppt1*^*−/−*^ microglia together (without any neurons present) suggest that these mutant astrocytes appear to drive these *Ppt1*^*−/−*^ microglia to become further activated (Fig. 10). Indeed, it seems plausible that the toxic effect of *Ppt1*^*−/−*^ microglia (Fig. 9) may be exacerbated by the consequence of impaired *Ppt1*^*−/−*^ astrocyte function and/or survival to create conditions that promote neuron loss, perhaps via the increased release of IL-1β from microglia (Additional file 1: Figure S1), but this awaits experimental verification. Previous data had suggested astrocyte activation was beneficial in this disorder (Macauley et al., 2011), since disease progression was accelerated in *GFAP*^*−/−*^*/Vimentin*^*−/−*^*/Ppt1*^*−/−*^ mice in which glial activation is genetically suppressed. This is in marked contrast to data from our co-culture systems that suggest a negative influence of *Ppt1*^*−/−*^ astrocytes upon neuron health (Figs. 8, 11), and it will be essential to validate these effects in vivo.

In order to address such issues, we are already generating mice in which Ppt1 can be inactivated in a cell type-specific manner. Such approaches would normally be complicated by the release of Ppt1 enzyme from genetically unmodified cells to cross correct the cell type in which Ppt1 has been inactivated. However, the generation of mice expressing a biologically active membrane tethered Ppt1 enzyme that is not secreted [42] provides important proof of principle for this strategy, which will be employed in our future studies. Nevertheless, this in vitro study has provided novel evidence for a greater extent of dysfunction in *Ppt1*^*−/−*^ glial cells than was previously anticipated and highlights the need for targeting glial cells in developing therapies for CLN1 disease. Gene therapy using newer generations of adeno-associated viruses has shown increasing efficacy, especially if these are targeted simultaneous to the brain and spinal cord [43]. These vectors predominantly transduce neurons, and these would secret Ppt1 enzyme to cross correct Ppt1 deficient glia within the CNS and correct their defects [10, 15, 45]. Regardless of such cross-correction, the administration of anti-inflammatory compounds to *Ppt1*^*−/−*^ mice provides additional benefit above gene therapy alone [28], and greater therapeutic efficacy may yet be provided by testing other drugs of this type. In a clinical setting, such neuroimmunomodulatory or anti-inflammatory approaches may be of use either before gene therapy can be administered or as an adjunct to gene therapy.

The NCLs have been grouped together traditionally on the basis of certain broadly similar clinical and pathological themes, but as new data emerge it is becoming evident that this is an oversimplification. It is clear that cultured astrocytes and microglia generated from CLN1 (this study) and CLN3 mice [35] are dysfunctional and exert a negative influence upon neuron health and may ultimately contribute to neurodegeneration. However, as the CLN1 data in this study reveal, the nature of these events differs markedly between these two major forms of NCL. Perhaps this is not surprising given that these two forms of NCL are caused by mutations in genes that encode such different types of proteins, one a lysosomal hydrolase (CLN1/PPT1) and the other a transmembrane protein whose function remains obscure (CLN3). Nevertheless, these findings have direct implications for our understanding of the cellular pathogenesis of these disorders and in this respect, it will be important to extend these studies to other forms of NCL.

## Additional file

Additional file 1: **Figure S1**. Expression of Interleukin-1β is increased following stimulation of Ppt1 deficient (*Ppt1*^*−/−*^) microglia. Supernatant was collected from wild type (WT) and *Ppt1*^*−/−*^ microglial cultures kept under basal and stimulated conditions for 24 h. Release of cytokines linked to oxidative stress was assessed using an ELISA kit (Signosis) and calculating changes in expression between basal and stimulated conditions. Secretion of Tumor necrosis factor α (TNFα), Transforming Growth Factor β (TGFβ), Monocyte Chemoattractant Protein-1 (MCP-1), Interleukin (IL)-1α, IL-6, IL-10 and IL-12 was not statistically significant between WT and *Ppt1*^*−/−*^ microglia, however secretion of IL-1β was significantly higher in *Ppt1*^*−/−*^ cultures. (Data shown as Mean ± SEM using a t-test, *n* = 3). (TIF 2444 kb)
