# Supplementary figures and images for: Compromised astrocyte function and survival negatively impact neurons in infantile neuronal ceroid lipofuscinosis
Source: Acta Neuropathol Commun. 2018 Aug 8;6:74. doi: 10.1186/s40478-018-0575-4 (PMC6081811; doi:10.1186/s40478-018-0575-4)

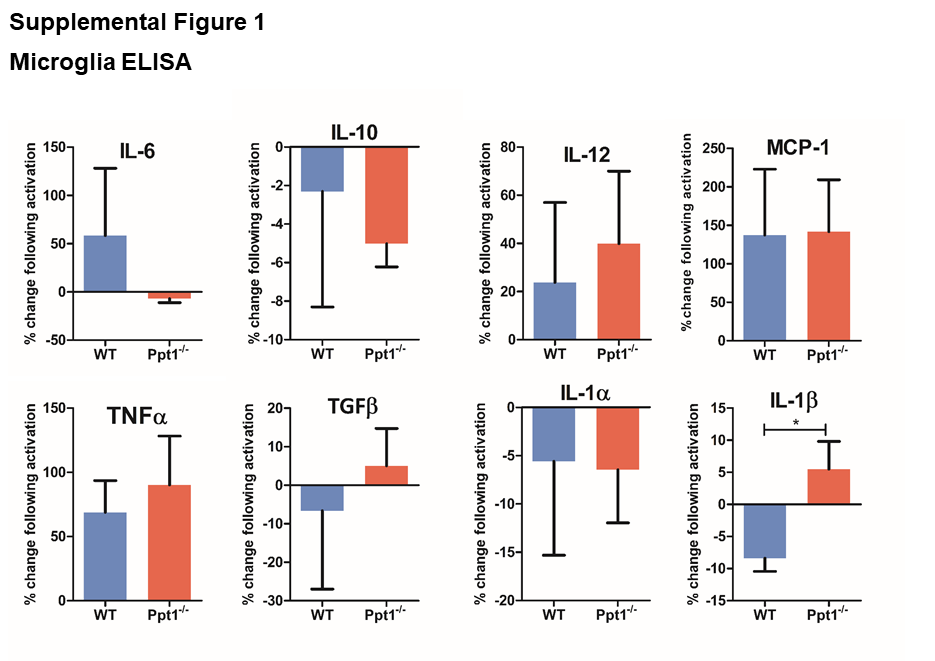

Supplement: Supplementary file 1 — Figure S1. Expression of Interleukin-1β is increased following stimulation of Ppt1 deficient (Ppt1−/−) microglia. Supernatant was collected from wild type (WT) and Ppt1−/− microglial cultures kept under basal and stimulated conditions for 24 h. Release of cytokines linked to oxidative stress was assessed using an ELISA kit (Signosis) and calculating changes in expression between basal and stimulated conditions. Secretion of Tumor necrosis factor α (TNFα), Transforming Growth Factor β (TGFβ), Monocyte Chemoattractant Protein-1 (MCP-1), Interleukin (IL)-1α, IL-6, IL-10 and IL-12 was not statistically significant between WT and Ppt1−/− microglia, however secretion of IL-1β was significantly higher in Ppt1−/− cultures. (Data shown as Mean ± SEM using a t-test, n = 3). (TIF 2444 kb) [file 40478_2018_575_MOESM1_ESM.tif]
